# The 7SK small nuclear ribonucleoprotein links the cell responses to transcription and replication stress by promoting replication fork reversal and homologous recombination

**DOI:** 10.1093/nar/gkag1021

**Published:** 2026-10-29

**Authors:** Akhil Bowry, Claire Wilson, Richard D W Kelly, Rosanna J Wilkins, Alexandra K Walker, Christy S Varghese, Zahraa A M Alshatti, Siobhan Murphy-Hollies, Joanna R Morris, Sylvain Egloff, Aditi Kanhere, Eva Petermann

**Affiliations:** Department of Cancer and Genomic Sciences, School of Medical Sciences, College of Medicine and Health, University of Birmingham, Birmingham B15 2TT, United Kingdom; Birmingham Centre for Genome Biology, University of Birmingham, Birmingham B15 2TT, United Kingdom; Department of Molecular and Clinical Cancer Medicine, Institute of Systems, Molecular and Integrative Biology, University of Liverpool, Liverpool L69 7BE, United Kingdom; Department of Cancer and Genomic Sciences, School of Medical Sciences, College of Medicine and Health, University of Birmingham, Birmingham B15 2TT, United Kingdom; Birmingham Centre for Genome Biology, University of Birmingham, Birmingham B15 2TT, United Kingdom; Department of Cancer and Genomic Sciences, School of Medical Sciences, College of Medicine and Health, University of Birmingham, Birmingham B15 2TT, United Kingdom; Birmingham Centre for Genome Biology, University of Birmingham, Birmingham B15 2TT, United Kingdom; Department of Cancer and Genomic Sciences, School of Medical Sciences, College of Medicine and Health, University of Birmingham, Birmingham B15 2TT, United Kingdom; Birmingham Centre for Genome Biology, University of Birmingham, Birmingham B15 2TT, United Kingdom; Department of Cancer and Genomic Sciences, School of Medical Sciences, College of Medicine and Health, University of Birmingham, Birmingham B15 2TT, United Kingdom; Birmingham Centre for Genome Biology, University of Birmingham, Birmingham B15 2TT, United Kingdom; Department of Cancer and Genomic Sciences, School of Medical Sciences, College of Medicine and Health, University of Birmingham, Birmingham B15 2TT, United Kingdom; Birmingham Centre for Genome Biology, University of Birmingham, Birmingham B15 2TT, United Kingdom; Department of Cancer and Genomic Sciences, School of Medical Sciences, College of Medicine and Health, University of Birmingham, Birmingham B15 2TT, United Kingdom; Birmingham Centre for Genome Biology, University of Birmingham, Birmingham B15 2TT, United Kingdom; Department of Cancer and Genomic Sciences, School of Medical Sciences, College of Medicine and Health, University of Birmingham, Birmingham B15 2TT, United Kingdom; Birmingham Centre for Genome Biology, University of Birmingham, Birmingham B15 2TT, United Kingdom; Centre de Biologie Intégrative de Toulouse, University of Toulouse, CNRS, CBI, Toulouse 31400, France; Department of Molecular and Clinical Cancer Medicine, Institute of Systems, Molecular and Integrative Biology, University of Liverpool, Liverpool L69 7BE, United Kingdom; Department of Pharmacology and Therapeutics, Institute of Systems, Molecular and Integrative Biology, University of Liverpool, Liverpool L69 7BE, United Kingdom; Department of Cancer and Genomic Sciences, School of Medical Sciences, College of Medicine and Health, University of Birmingham, Birmingham B15 2TT, United Kingdom; Birmingham Centre for Genome Biology, University of Birmingham, Birmingham B15 2TT, United Kingdom

## Abstract

The 7SK small nuclear ribonucleoprotein (7SK-snRNP) releases positive transcription elongation factor b to sustain RNA polymerase II (RNAPII) activity during transcription stress. Many conditions that block transcription also cause replication stress, and 7SK-snRNP components have potential roles in transcription–replication conflicts and double-strand break (DSB) repair. Here, we investigate roles of 7SK-snRNP components in replication stress responses. We report that 7SK-snRNP promotes replication fork slowing in response to agents that cause both replication and transcription stress. Our data suggest that 7SK-snRNP facilitates RAD51-mediated replication fork reversal rather than transcription–replication conflicts. 7SK-snRNP also promotes RAD51 recruitment to replication-associated DSBs or post-replicative gaps under conditions of transcription stress and supports homologous recombination at direct DSBs. In contrast, 7SK-snRNP components are not required for RAD51 recruitment in response to hydroxyurea. Our data support that LARP7 functions during replication stress independently of its interaction with BRCA1, and that LARP7 and HEXIM1 promote survival of replication stress. 7SK-snRNP components are not recruited to stressed replication forks, and RNAPII inhibition phenocopies loss of these proteins. Our data support a model where 7SK-snRNP modulation of RNAPII activity helps facilitate several RAD51 functions under transcription stress conditions, thereby connecting the cell responses to transcription and replication stress.

## Graphical Abstract

**Graphical Abstract:**
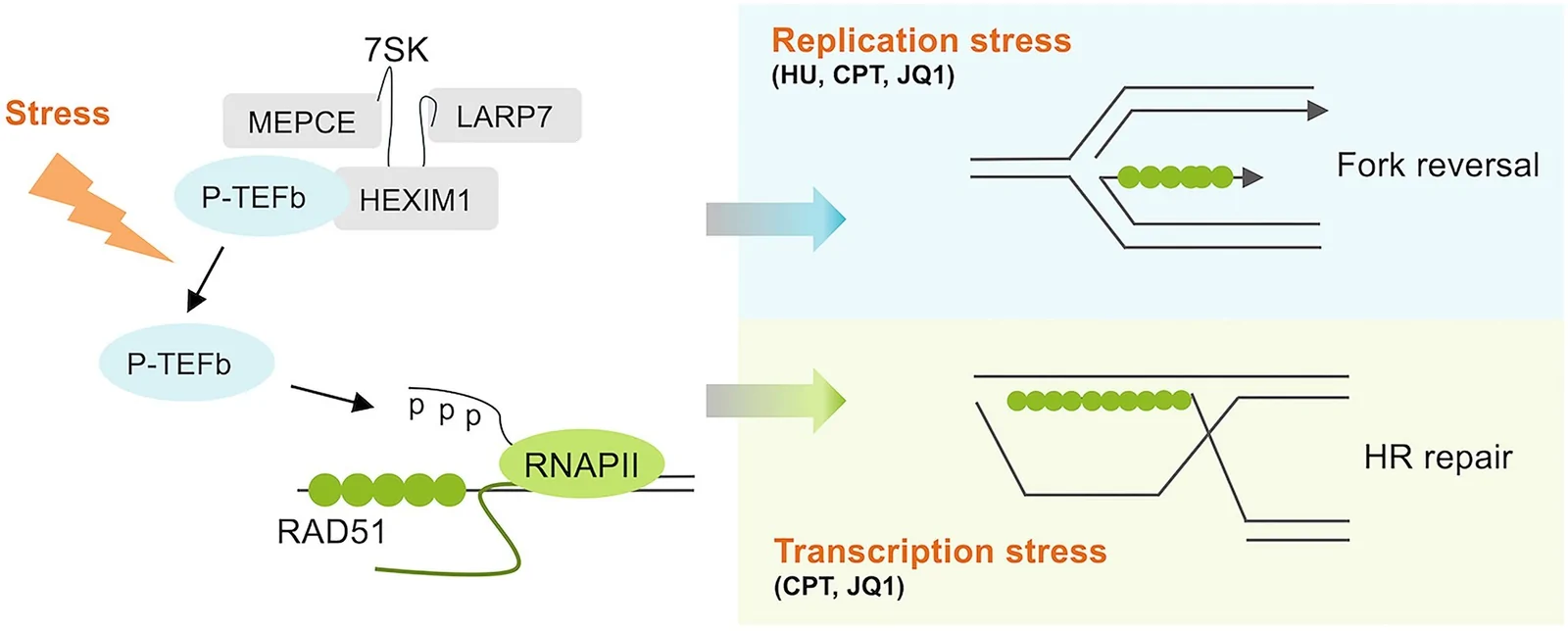

## Introduction

Cancer therapies induce cellular stress to kill cancer cells while relying on altered proliferation and stress responses in cancer versus normal cells for specificity. Many cytotoxic cancer therapies damage DNA, causing replication stress and activating DNA repair [1]. DNA damage can also block transcription, leading to transcription stress, defined as RNA polymerase II (RNAPII) pausing, stalling, or backtracking [2]. It is widely appreciated that replication stress and cellular responses to replication stress play important roles in cancer development and treatment [3]. How cellular replication stress responses interplay with transcription stress in cancer has received less attention.

Responses to transcription stress include activation of transcription-coupled nucleotide excision repair and degradation of stalled RNAPII [2]. Another important player in transcription stress is the 7SK small nuclear ribonucleoprotein (7SK-snRNP), which modulates RNAPII activity in response to a wide variety of treatments that target transcription, including UV radiation and other inducers of bulky DNA adducts, platinum compounds, topoisomerase I (TOP1) inhibitors, and inhibitors of epigenetic regulators such as HDACs, BET proteins, and DNA methyltransferases [4–8].

The 7SK-snRNP is comprised of the non-coding 7SK RNA and the RNA-binding proteins LARP7, MEPCE, and HEXIM1 [9, 10]. The complex binds and inactivates the positive transcription elongation factor b (P-TEFb). Signalling through stress-activated pathways such as p38 MAPK causes 7SK-snRNP to release active P-TEFb [8, 9], which promotes promoter-proximal pause release of RNAPII by phosphorylating RNAPII and the transcription elongation factors DSIF and NELF [11] (Fig. 1A). Recent work has highlighted that this process is required for a rapid burst in transcription, despite the presence of transcription-blocking DNA damage, of short stress response and non-coding RNA genes that promote survival through largely unknown mechanisms [7, 8]. 7SK-snRNP loss and P-TEFb inhibitors both suppress synthesis of stress-induced transcripts, suggesting that a main stress response function of 7SK-snRNP is not its role as P-TEFb inhibitor, but its role in releasing additional active P-TEFb [7, 8].

**Figure 1.**
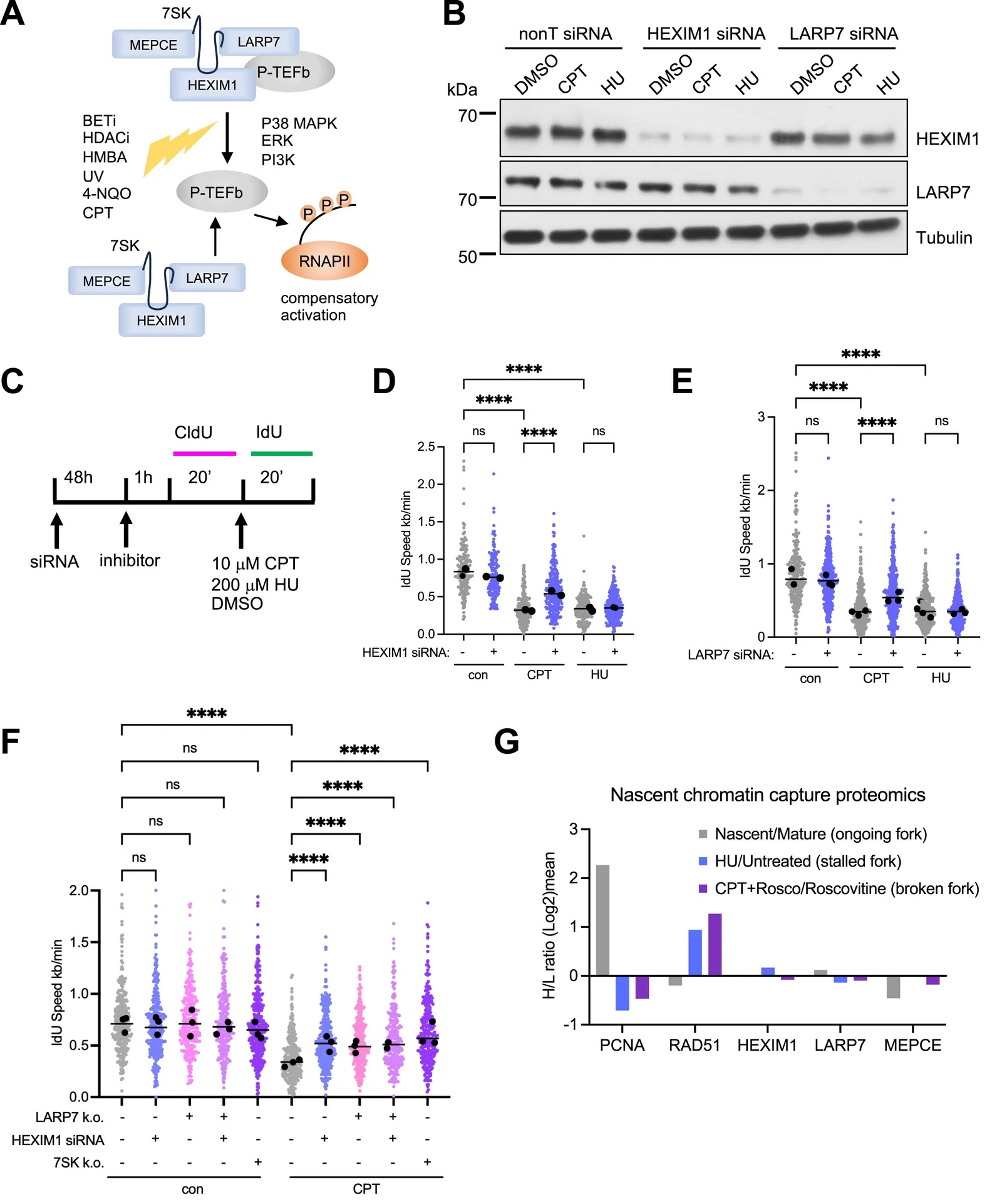
7SK-snRNP components promote fork slowing induced by camptothecin (CPT), but not hydroxyurea (HU). (**A**) Schematic of active P-TEFb release from 7SK-snRNP to promote RNAPII pause release in response to stress and growth stimuli. (**B**) Protein levels of HEXIM1, LARP7, and Tubulin (loading control) in U2OS cells 48 h after small interfering RNA (siRNA) transfection. nonT = non-targeting control siRNA. (**C**) Approach for siRNA depletion or small molecule inhibitor treatment prior to DNA fibre labelling in presence of CPT (10 mM) or HU (200 mM). (**D**) Replication fork speeds (IdU label) after CPT or HU treatment in presence of HEXIM1 (+) or nonT (−) siRNA. Data from 2 repeats. (**E**) Replication fork speeds (IdU label) after CPT or HU treatment in presence of LARP7 (+) or nonT (−) siRNA. Data from 2–3 repeats. (**F**) Replication fork speeds (IdU label) after CPT treatment in wild type (WT), LARP7 KO, or 7SK KO HAP1 cells transfected with nonT siRNA or HEXIM1 siRNA as indicated. Data from 3 repeats (*LARP7* k.o. + HEXIM1 siRNA and CPT: 2 repeats). (**G**) Nascent chromatin capture proteomics in HeLa S3 cells [40] shows limited enrichment or depletion of HEXIM1, LARP7, and MEPCE at ongoing (nascent/mature chromatin), stalled (3 mM HU, 30 min/untreated), or collapsed (1 μM CPT, 40 min + roscovinite/roscovitine) replication forks compared to PCNA or RAD51. Roscovitine was included to prevent new origin firing. Scatter graphs show aggregates and medians (black points) of independent repeats with overall median (line). Kruskal–Wallis with multiple comparisons test, ^****^P ≤ .0001; ns: not significant.

The control of P-TEFb by 7SK-snRNP has been proposed as an anti-cancer mechanism preventing epithelial-mesenchymal transition and metastasis [12]. LARP7 and HEXIM1, which are crucial for the function of the 7SK-snRNP, are potential tumour suppressors whose protein levels are decreased in leukaemia and in solid cancers [13]. Such alterations in 7SK-snRNP components could impact the response to transcription blocking cancer treatments.

The transcription-targeting or -blocking agents that induce release of P-TEFb from the 7SK-snRNP frequently also cause replication stress characterized by replication fork slowing. Unresolved replication stress can lead to DNA damage such as single-stranded gaps (ssDNA gaps) or single-ended double-strand breaks (seDSBs), which are implicated in agent toxicity [3]. Homologous recombination (HR) repair, which depends on RAD51, helps repair ssDNA gaps and seDSBs [14, 15] and is an important determinant of the response to replication stress-inducing cancer therapies [1]. HR factors such as RAD51 also prevent or resolve conflicts between transcription and replication [16, 17], and transcription promotes HR at DSBs [18]. Transcription and replication, as a source of potential conflicts leading to replication stress and DNA damage, are increasingly understood as intertwined in cancer.

In light of this, we previously reported that the 7SK-snRNP component HEXIM1 is required for increased RNA synthesis and RAD51-dependent replication fork slowing in response to the BET inhibitor JQ1 [19]. This suggested a role for HEXIM1 in promoting transcription–replication conflicts, but paradoxically, HEXIM1 also prevented DNA damage induction in JQ1-treated cells [19]. Others have since implicated LARP7 or MEPCE in preventing transcription–replication conflicts in absence of BRCA1, and in modulating HR repair at DSBs [20–22]. However, while LARP7 is proposed to prevent HR, MEPCE is proposed to promote HR [20, 22].

Here, we aimed to investigate the wider roles of the 7SK-snRNP in the response to replication stress induced by TOP1 inhibitor CPT or ribonucleotide reductase inhibitor HU. CPT stabilizes TOP1 cleavage complexes, within minutes causing single-strand breaks [23], torsional stress [24], RNAPII stalling downstream of RNAPII pause sites, genome-wide changes in RNA:DNA hybrid occupancy, and replication-dependent seDSBs [25, 26]. HU causes reactive oxygen species and deoxyribonucleotide triphosphate depletion or imbalance, which both slow and stall replication forks [27, 28].

We report that HEXIM1 and LARP7 promote replication fork slowing in response to agents that cause both replication and transcription stress, in a manner consistent with their canonical 7SK-snRNP functions. Our data support that this role in fork slowing is mainly through facilitating RAD51-mediated replication fork reversal. HEXIM1 and LARP7 also promote RAD51 recruitment to DNA damage sites, HR repair, and cell survival, especially under conditions of transcription stress such as induced by CPT, suggesting that the 7SK-snRNP links the cell responses to transcription and replication stress.

## Materials and methods

### Cell lines and reagents

U2OS and HeLa cells were obtained from ATCC. HCT116 cells were obtained from Charles Swanton. U2OS-DR-GFP cells were generously provided by Jeremy Stark (City of Hope, Duarte USA). HAP1 cells were purchased from Horizon Discovery. The generation and characterization of HAP1 LARP7 KO and 7SK KO are described in [7]. U2OS and HCT116 cells were authenticated by STR profiling (ATCC). Cells were confirmed to be free of Mycoplasma infection by polymerase chain reaction (PCR) and grown in a humidified atmosphere containing 5% CO_2_. U2OS, HCT116, and HeLa cells were grown in Dulbecco’s modified Eagle’s Medium with 10% foetal bovine serum (FBS), L-Glutamine (2 mM), Streptomycin (50 μg/ml) and Penicillin (50 U/ml). HAP1 cells were grown in Iscove’s Modified Dulbecco’s Medium supplemented with 10% FBS, sodium pyruvate (1 mM), and penicillin and streptomycin (100 mg/ml).

CPT, HU, hexamethylene bisacetamide (HMBA, 5 mM), 5,6-Dichlorobenzimidazole 1-β-D-ribofuranoside (DRB, 100 μM), and REV1 inhibitor JH-RE-06 (2.5 μM) were from Sigma–Aldrich. (+)-JQ1 was from Tocris Bioscience. P38 MAPK inhibitor (SB203580, Startech Scientific 10 μM) was from Selleckchem. Mirin (50 μM) was from Sigma–Aldrich.

### CDK9 salt fractionation

U2OS cells were harvested after treatments before being washed twice in cold phosphate buffered saline (PBS). Cells were lysed in low-salt buffer [10 mm KCl, 10 mm MgCl_2_, 10 mm HEPES, pH 7.5, 1 mm ethylenediaminetetraacetic acid (EDTA), 1 mm Dithiothreitol (DTT), 0.5% Nonidet *P*-40, and protease inhibitor cocktail] before being incubated on ice for 10 min. Lysates were centrifuged 5000 × *g* for 5 min, and the supernatant was extracted, which represented the soluble fraction. The pellets were washed in low-salt buffer and then resuspended in high-salt buffer (450 mm NaCl, 1.5 mm MgCl_2_, 20 mm HEPES, pH 7.5, 0.5 mm EDTA, 1 mm DTT, 0.5% Nonidet *P*-40, and protease inhibitor cocktail) and incubated on ice for 10 min. Samples were then spun down, and suspensions were collected, which corresponded with the chromatin pellet (CP) fraction.

### siRNA and DNA transfection

ON-TARGET siRNA SMARTpools against LARP7 (L-020996-01-0005), HEXIM1 (L-012225-01-0005), and BRCA1 (L-012225-01, L-003461-00), siGenome SMARTpool against MUS81 (D-016143), siGenome siRNA against ZRANB3 (D-010025-03), and custom siRNAs against RAD51 [29], CtIP [30], and PRIMPOL [31] were from Dharmacon/Horizon Discovery, and ‘Allstars negative control siRNA’ was from Qiagen. Cells were plated in six-well plates and transfected the next day with 50 nM siRNA using Dharmafect-1 reagent (Horizon Discovery). For combined siRNA and plasmid transfection, cells were plated in six-well plates and transfected the next day with 2.5 μg plasmid and 50 nM siRNA using Transit-X2 (Mirus Bio). pcDNA3.1(+)-MYCtag-HEXIM1-WT-siRNA-resistant and pcDNA3.1(+)-MYCtag-HEXIM1-Y203D-T205D-siRNA-resistant were synthesized by GenScript with the HEXIM1 coding sequence modifications listed in Supplementary Table S2. For RNase H1 overexpression, cells were plated in six-well plates at 70% confluency and transfected the next day using TransIT-2020 (Mirus Bio) with 2.5 μg pCMV6-AC-RNase H1-GFP (Origene) or control plasmid pEGFP-C2 (Clontech).

### Western blotting

Cells were lysed by pipetting up and down in UTB buffer (50 mM Tris–HCl, pH 7.5, 150 mM β-mercaptoethanol, and 8 M urea), and lysates were sonicated twice for 10 s each to release DNA-bound proteins. Proteins were separated by sodium dodecyl sulphate (SDS)–polyacrylamide gel electrophoresis before being transferred to a nitrocellulose membrane. Primary antibodies used were monoclonal rabbit anti-HEXIM1 (Abcam ab25388,1:5000), anti-LARP7 (Abcam, ab134757,1:1000), rabbit anti-CDK9 (Cell Signalling C12F7. 1:1 000), rabbit anti-Turbo-GFP to detect GFP-RNase H1 (Evrogen AB513, 1:10 000), rabbit anti-ZRANB3 (Proteintech 23111-1-AP, 1:500), rabbit anti-RAD51 (Abcam, ab63801, 1:1 000), mouse MUS81 (Novus Biologicals NB1000-2064, 1:500), rabbit anti-MYC-tag (Cell Signaling 2272, 1:500), mouse anti-αTUBULIN (Sigma–Aldrich T6074, 1:10 000), and rabbit anti-βACTIN (Cell Signaling 4967, 1:5000). The membrane was washed before being probed with HRP-linked secondary antibodies. Detection was performed using ECL detection or ChemiDoc™ MP Imaging System (Bio-Rad) and quantified using ImageJ.

### DNA fibre analysis

U2OS cells were pulse-labelled with 25 mM CldU and 250 mM IdU, where treatments with CPT or HU were added at the same time with IdU as indicated. Cells were harvested, and DNA fibre spreads were performed mixing cell sample with spreading buffer (200 mM Tris, pH 7.4, 50 mM EDTA, 0.5% SDS) to lyse cells before being spread down the slide. Fibre spreads were denatured with 2.5 M HCl for 1 h 15 min, incubated with rat anti-BrdU (BU1/75, Abcam ab6326, 1:700) and mouse anti-BrdU (B44, Becton Dickinson 347580, 1:500) for 1 h, fixed with 4% paraformaldehyde (PFA), and incubated with anti-rat AlexaFluor 594 and anti-mouse AlexaFluor 488 (Invitrogen, 1:500) for 1.5 h before mounting in Fluoroshield mounting medium (Sigma–Aldrich). Fibers were examined using a Nikon E600 microscope with a Nikon Plan Apo 60× (1.3 NA) lens, an Andor digital camera, and the NIS-Elements acquisition software (Nikon Instruments). For quantification of fork speeds, the lengths of labelled tracks were measured using the ImageJ software [32] (http://rsbweb.nih.gov/ij/).

### Immunofluorescence

Cells were fixed with 4% PFA for 10 min and permeabilized with 0.25% Triton X-100 for 5 min at 4°C, followed by blocking with 2% bovine serum albumin (BSA), 0.2% Tween 20 in PBS. Primary antibodies were rabbit polyclonal anti-RAD51 (Abcam ab63801, 1:500), mouse monoclonal anti-phospho-Histone H2AX (Ser139) (JBW301, Millipore, 1:1000), and rabbit polyclonal anti-53BP1 (Bethyl A300-272A, 1:15 000). Secondary antibodies were anti-rabbit IgG AlexaFluor 594 and anti-mouse IgG AlexaFluor 488 (Thermo Fisher). For RAD51 foci detection, cells were also incubated with 1 μM EdU for 30 min to mark replicating cells, followed by Click reaction according to the Click-iT EdU Imaging Kit (Thermo Fisher) manufacturer’s instructions. Coverslips were mounted in Fluoroshield with 4′,6-diamidino-2-phenylindole (DAPI) (Sigma–Aldrich). DNA was counterstained with DAPI, and slides were examined using a Nikon E600 microscope as before. Cells with >5 RAD51 foci and 8 γH2AX or 10 53BP1 foci were scored as positive.

### EU incorporation assay

EU incorporation was performed using the Click-It RNA Alexa Fluor 594 Imaging kit (Thermo Scientific). Cells were incubated with 1 mM EU as indicated, fixed, and then permeabilized. Click reactions were performed according to manufacturer’s instructions, except that Hoechst staining was omitted and coverslips were instead mounted in Fluoroshield with DAPI (Sigma–Aldrich). Images were acquired using a Nikon E600 microscope and NIS-Elements acquisition software as before. ImageJ was used to generate a DAPI nuclear mask, and the mean AlexaFluor 594 intensity per pixel was quantified per nucleus.

### Chromatin RNA-seq

Cells were plated in equal numbers before being treated and harvested. The cells were then resuspended in cytoplasmic lysis buffer (0.15% NP-40, 10 mM Tris–HCl, pH 7.0, 150 mM NaCl) before being incubated on ice for 5 min. The lysate was then poured on top of sucrose buffer (10 mM Tris–HCl, pH 7.0, 150 mM NaCl, 25% sucrose) and centrifuged at 16 000 × *g* for 10 min. The pellet represents the nuclei, and the supernatant represents the cytosolic fraction, which was removed and kept. The nuclei were resuspended in nuclei wash buffer (0.1% Triton X-100, 1 mM EDTA, in 1× PBS) before being centrifuged at 1500 × *g* for 1 min. The pellet was resuspended in glycerol buffer (20 mM Tris–HCl, pH 8.0, 75 mM NaCl, 0.5 mM EDTA, 50% glycerol, 0.85 mM DTT), and then nuclei lysis buffer (1% NP-40, 20 mM HEPES, pH 7.5, 300 mM NaCl, 1 M urea, 0.2 mM EDTA, 1 mM DTT) was added before the sample was vortexed and put on ice. The sample was centrifuged further for 2 min at 18 500 × *g*, and the nucleoplasm fraction was extracted by removing the supernatant. The chromatin was resuspended in chromatin resuspension buffer (25 μM α-amanitin, 50 Units SUPERaseIN, Protease inhibitors cOmplete in 1× PBS) before being stored at −80°C. The chromatin fraction was extracted using the Zymol RNA extraction kit following manufacturer’s instructions before the RNA was quantified and stored at −80°C.

Chromatin RNA sequencing (RNA-seq) was performed on chromatin fractions. Chromatin RNA-seq libraries were prepared using NEBNext Ultra II 8482 Directional RNA Library Prep kit for Illumina (New England Biolabs), and all libraries were paired-end sequencing on NovaSeq 6000 (Illumina). Sequences were first quality checked using FastQC(v0.11.9), (https://www.bioinformatics.babraham.ac.uk/projects/fastqc/), then quality filtered and trimmed using TrimGalore (v0.6.6). (https://www.bioinformatics.babraham.ac.uk/projects/trim_galore/). Genome mapping was performed with STAR (v2.7.10b) [33] against the human reference genome (hg38 assembly). Samtools (v1.13) [33] was used to retain only properly paired and mapped reads. Strand-specific bam files were created using samtools (https://www.htslib.org/), with strand-specific bigwig files created using BEDtools (v2.30.0) [34] genome coverage functions. Counts for genes and genomic regions were calculated using samtools [33] bedcoverage with GENCODE v43 gene annotation [35]. RPKM and LogFC values were calculated using R Studio (v4.4.0) [36], with differential genes identified by LogFC > 2. Upregulated and downregulated genes were classified as those genes which were upregulated or downregulated in all three replicates. Gene Ontology of up- and downregulated genes was performed using g:Profiler [37]. Profile plots and heatmaps were generated using deepTools (v3.5.6) [38].

### Reverse transcription quantitative real-time PCR

Cells were harvested in TRI Reagent® (Zymo Research), and RNA extraction was performed using the Direct-zol RNA Miniprep Kit (Zymo Research), including DNase I treatment, according to the manufacturer’s instructions. RNA quality and concentration were determined using the 4200 TapeStation (Agilent) or a NanoDrop Lite spectrophotometer (Thermo Fisher). Total RNA (250 ng – 1 mg) was reverse-transcribed using a mix of poly-A and random hexamer primers and the qScript complementary DNA (cDNA) synthesis kit (Quantabio) or Tetro™ cDNA synthesis kit (Meridian Bioscience), following the manufacturer’s instructions. For transcription in the DR-GFP reporter, ProtoScript® First Strand cDNA Synthesis Kit (NEB) and site-specific primers were used. Primers were from Merck (Supplementary Table S3). Two microlitres cDNA and primers were added to SYBR™ Green PCR Master Mix (Thermo Fisher) and analysed using the Real Time PCR QuantStudio 5 system (Thermo Fisher). Cycling parameters were 95°C/5 min, 40 cycles of 95°C/15–30 s, annealing (temperatures see Supplementary Table S3) for 15–30 s, and 72°C/15–30 s. DCt values were normalized to *ACTB*, or *RPLP0* for transcription in the DR-GFP reporter.

### Slot blot

Genomic DNA was extracted using the DNeasy Blood & Tissue kit (Qiagen). Ten micrograms genomic DNA per sample was treated with 2 U/µg DNA of RNaseH (NEB, M0297), or mock treated, for 2 h at 37°C. Two hundred fifty nanograms per well was loaded onto the slot blot apparatus, transferred onto pre-wetted nylon membrane (Amersham Hybond N+). After UV crosslinking at 1200 µJ and blocking in sterile 5% milk/TBST for 1 h at room temperature, the membrane was incubated overnight at 4°C in mouse anti-DNA-RNA hybrid antibody (S9.6 hybridoma growth medium, ATCC HB-8730, 1:1000) or mouse anti-double-stranded DNA (dsDNA) antibody (Abcam ab27156, 1:100 000) in sterile 5% BSA/TBST, before being washed in TBST and incubated in goat anti-mouse HRP (Cell Signaling 7074, 1:5 000) 5% milk/TBST for 1 h at room temperature. Images were collected using a ChemiDoc™ MP Imaging System (Bio-Rad) and quantified using ImageJ.

### Homologous recombination assay

U2OS-DR-GFP cells and expression constructs for RFP and I-Sce I endonuclease were generously provided by Jeremy Stark (City of Hope, Duarte, USA). U2OS-DR3-GFP cells were transfected with the indicated siRNAs using Dharmafect1 (Dharmacon). After 24 h, growth medium was replaced, and cells were co-transfected with RFP and I-Sce I constructs using FuGene6 (Promega) or *Trans*IT^®^-2020 (Mirus). Twenty-four hours later, the medium was replaced, and cells were cultured for an additional 48 h before fixation in 2% PFA in PBS. GFP-positive cells were detected on a Fortessa X-20 flow cytometer and analysed with the BD FacsDiva software. A total of 20 000 cells were acquired per sample, and the percentage of GFP-positive cells was determined as a fraction of RFP positive cells.

### Propidium iodide flow cytometry

Cells were fixed in 70% ethanol at −20°C for 30 min, then centrifuged at 500 × *g* for 5 min. After two washes in PBS, cells were mixed with PBS containing 10 µg/ml propidium iodide and 25 µg/ml ribonuclease A (Merck). Cell cycle profiles were generated and analysed using the Fortessa X-20 flow cytometer and BD FacsDiva software, respectively.

### Colony survival assay

Forty-eight hours after siRNA transfection, U2OS cells were trypsinized and plated in triplicate at a density of 800–2000 cells per well. Cells were treated with drugs continuously until colonies of >50 cells had grown, then stained with 50% ethanol, 2% methylene blue, and colonies counted. IC50 values were calculated using the built-in nonlinear regression (curve fit) function in GraphPad Prism. The equation used was [Inhibitor] versus normalized response – Variable slope, least squares fit.

### Quantification and statistical analysis

Values represent the means + 1× SEM of at least three independent biological repeats. The number of independent biological repeats (*n*) is indicated in the figure legends. For foci analysis, at least 10 different areas were quantified for each independent biological repeat. Gaussian distribution was determined, and statistical tests were performed using the GraphPad Prism 10 software, version 10.2.0 (392). For comparing two datasets with non-Gaussian distribution, Mann–Whitney test was used. For multiple comparisons, one-or two-way analysis of variance (ANOVA) or mixed-effects analysis with Dunnett’s, Tukey’s, or Sidak’s test was used for datasets with Gaussian distribution, and the Kruskal–Wallis test with Dunn’s test for datasets with non-Gaussian distribution.

## Results

### The 7SK-snRNP promotes replication fork slowing in response to CPT, but not HU

To investigate functions of the 7SK-snRNP in replication stress, we used siRNA to deplete human U2OS osteosarcoma cells of HEXIM1 or LARP7 (Fig. 1B). Loss of 7SK-snRNP components prevents the DNA damage-induced release of additional P-TEFb [7]. HEXIM1 or LARP7 depletion was combined with DNA fibre analysis to investigate whether loss of 7SK-snRNP components alters replication fork progression in presence of replication stress-inducing agents. Cells were labelled with CldU to mark ongoing forks, followed by IdU combined with CPT or HU to measure replication fork progression during drug treatment (Fig. 1C). The concentrations of both drugs were chosen to reduce replication fork speeds to a similar extent, as higher HU concentrations would block replication fork progression (Fig. 1D). As we previously observed for JQ1 [19], HEXIM1 depletion partially rescued replication fork slowing induced by CPT. In contrast, HEXIM1 depletion did not rescue replication fork slowing induced by HU (Fig. 1D). While CPT induces many more DSBs than HU, HEXIM1 depletion had also rescued fork slowing induced by JQ1, which does not induce DSBs [19]. A similar partial rescue of treatment-induced fork slowing was observed when using lower CPT concentrations (Supplementary Fig. S1A), or when LARP7 was depleted (Fig. 1E). CldU fork speeds were unaffected by HEXIM1 or LARP7 depletion (Supplementary Fig. S1B and C).

We next asked whether CPT-induced replication fork slowing requires the entire 7SK-snRNP complex. We used HAP1 cell lines with CRISPR-Cas9 inactivation of either the *LARP7* or *RN7SK* gene (LARP7 KO and 7SK KO), which causes loss of the 7SK RNA and therefore loss of the 7SK-snRNP [7]. Inactivation of *LARP7* or *RN7SK* partially rescued fork slowing in response to CPT and did so to a similar extent as HEXIM1 siRNA depletion either in HAP1 WT or in LARP7 KO cells (Fig. 1F). This supports that HEXIM1 and LARP7 rescue fork slowing by acting in the same pathway, and by acting as components of the 7SK-snRNP. To investigate whether the 7SK-snRNP is recruited to stressed replication forks, we interrogated published replication fork proteome data. Proteomics studies using iPOND [39] or nascent chromatin capture combined with stable isotope labelling by amino acids in cell culture [40] (Fig. 1G) did not show enrichment of HEXIM1, LARP7, or MEPCE at ongoing, stalled, or collapsed replication forks, which suggests that these proteins do not act directly at forks.

### CPT-induced fork slowing requires P-TEFb regulation by 7SK-snRNP

The 7SK-snRNP is required for the release of active P-TEFb in response to stress. Under transcription stress conditions, 7SK-snRNP loss, preventing P-TEFb release, and P-TEFb inhibition have similar effects [7, 8]. We therefore asked whether other approaches to preventing P-TEFb release and activation could also rescue CPT-induced fork slowing. We inhibited p38 MAPK, which has been implicated in promoting release of P-TEFb from the 7SK-snRNP [8], with p38 inhibitor SB203580, or CDK9, the catalytic subunit of P-TEFb itself, with 5,6-Dichlorobenzimidazole 1-β-D-ribofuranoside (DRB) (Fig. 2A). While CDK9 inhibition itself causes transcription stress and releases P-TEFb from the 7SK-snRNP [41], any impact on P-TEFb activity would be counteracted by the simultaneous inhibition of CDK9. Both p38 inhibition and CDK9 inhibition partially rescued replication fork slowing induced by CPT but did not rescue fork slowing induced by HU (Fig. 2B and C). Both DRB treatment and HEXIM1 depletion prevented CPT-induced fork slowing to the same extent, whether alone or in combination (Fig. 2D). This supports that, as reported by others [8], P-TEFb inhibition phenocopies the effect of losing 7SK-snRNP components.

**Figure 2.**
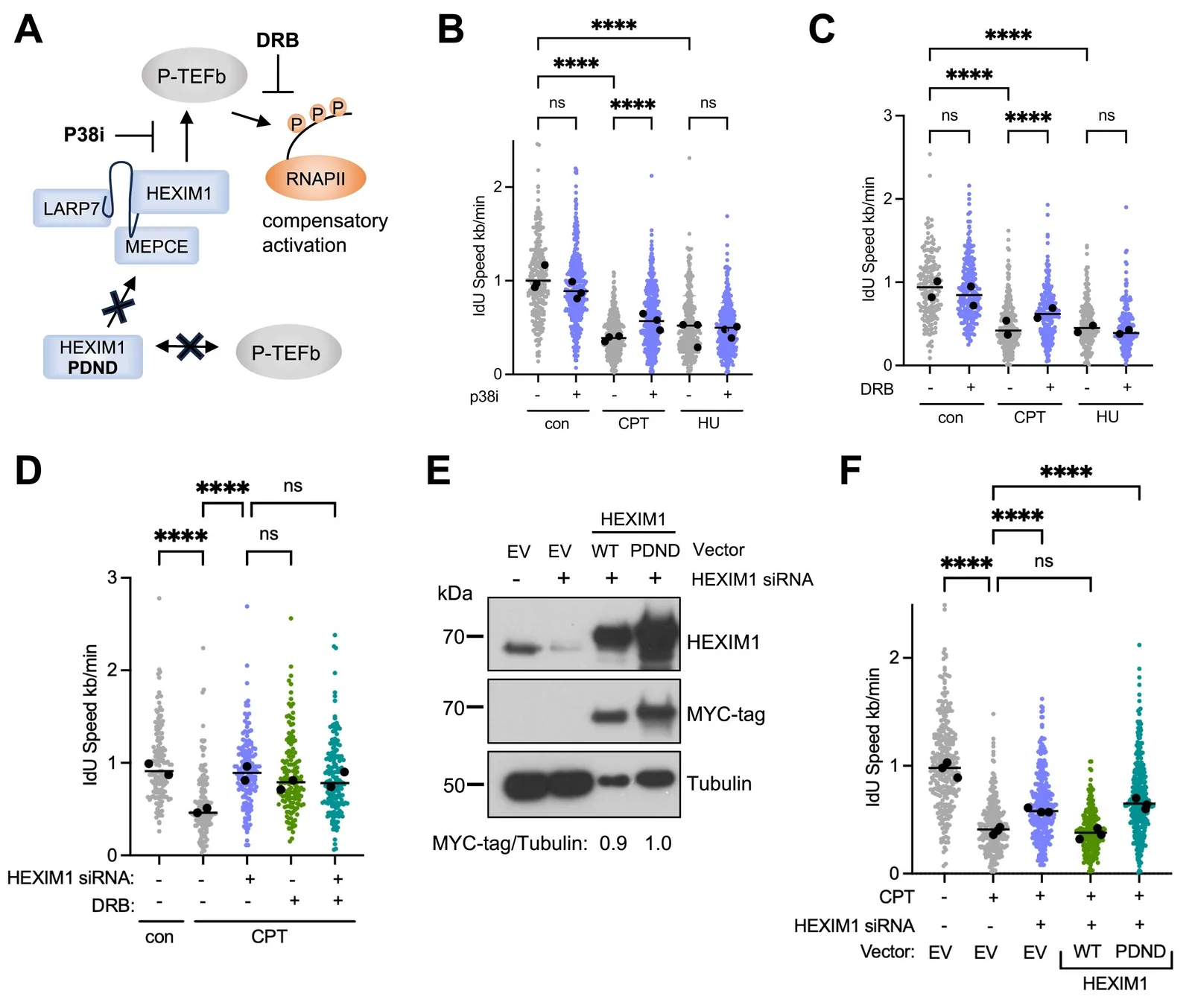
P-TEFb regulation by 7SK-snRNP promotes fork slowing. (**A**) DRB inhibits P-TEFb, and p38 MAPK inhibitor (P38i) inhibits 7SK-snRNP/P-TEFb dissociation. The HEXIM1 PDND mutant cannot interact with P-TEFb or 7SK RNA. (**B**) Replication fork speeds (IdU label) after CPT or HU treatment ± p38i. Data from three repeats. (**C**) Replication fork speeds (IdU label) after CPT or HU treatment ± 100 μM DRB. Data from two repeats. (**D**) Replication fork speeds (IdU label) after CPT treatment ± DRB in presence of nonT (−) or HEXIM1 (+) siRNA. Data from two repeats. (**E**) Protein levels of HEXIM1, MYC-tag, and Tubulin (loading control) in U2OS cells 48 h after transfection with nonT or HEXIM1 siRNA and empty vector (EV) or plasmids encoding WT or mutant (PDND) HEXIM1. (**F**) Replication fork speeds (IdU label) after CPT treatment 48 h after transfection with nonT (−) or HEXIM1 (+) siRNA and EV or plasmids encoding WT or PDND HEXIM1. Data from three repeats. Scatter blots show aggregates and medians (black points) of independent repeats with overall median (line). Column graphs show mean ± SEM. Kruskal–Wallis with multiple comparisons test, ***P ≤ .001; ^****^P ≤.0001; ns: not significant.

To further decipher whether the P-TEFb-binding function of HEXIM1 was required for HEXIM1-dependent fork slowing, we ectopically expressed WT and mutant HEXIM1. Cells were transfected with HEXIM1 siRNA and control plasmid or plasmids encoding siRNA-resistant, MYC-tagged WT HEXIM1 or the HEXIM1 Y203D/T205D (PDND) mutant, which cannot interact with either P-TEFb or the 7SK RNA [42, 43] (Fig. 2E). WT HEXIM1, but not the PDND mutant, restored CPT-induced fork slowing (Fig. 2F). Finally, HMBA, a different drug that releases P-TEFb from 7SK-snRNP [44], also caused HEXIM1-dependent fork slowing (Supplementary Fig. S1D).

Taken together, these data support that the 7SK-snRNP complex and its release of active P-TEFb promote replication fork slowing in presence of CPT, as well as JQ1 and HMBA.

### CPT, but not low-dose HU, activates a transcription stress response

We next compared the impact of CPT, JQ1, and HU on transcription stress and P-TEFb dynamics. To measure the relocation of P-TEFb, cells were fractionated into a soluble (cytoplasmic and nucleoplasmic) fraction by extracting whole cells with low-salt buffer conditions, and a chromatin fraction by extracting CPs with high-salt buffer conditions [41]. P-TEFb moves from the soluble nucleoplasmic into the chromatin fraction in response to CPT, which correlates with P-TEFb release from the 7SK-snRNP [5]. Fractions were probed for CDK9 using western blot (Fig. 3A and B). Quantification of CDK9 signal intensity followed by normalization to control demonstrated that as expected based on previous reports [4, 5], both JQ1 and CPT treatment increased P-TEFb in the chromatin fraction. In contrast, HU treatment slightly increased the fraction of P-TEFb in the soluble fraction (Fig. 3C and Supplementary Fig. S2A). Reverse transcription quantitative real-time PCR (RT-qPCR) showed that expression of transcription stress-responsive genes *SERTAD1* and *EGR1* [8, 19] increased in response to CPT, but not HU (Fig. 3D).

**Figure 3.**
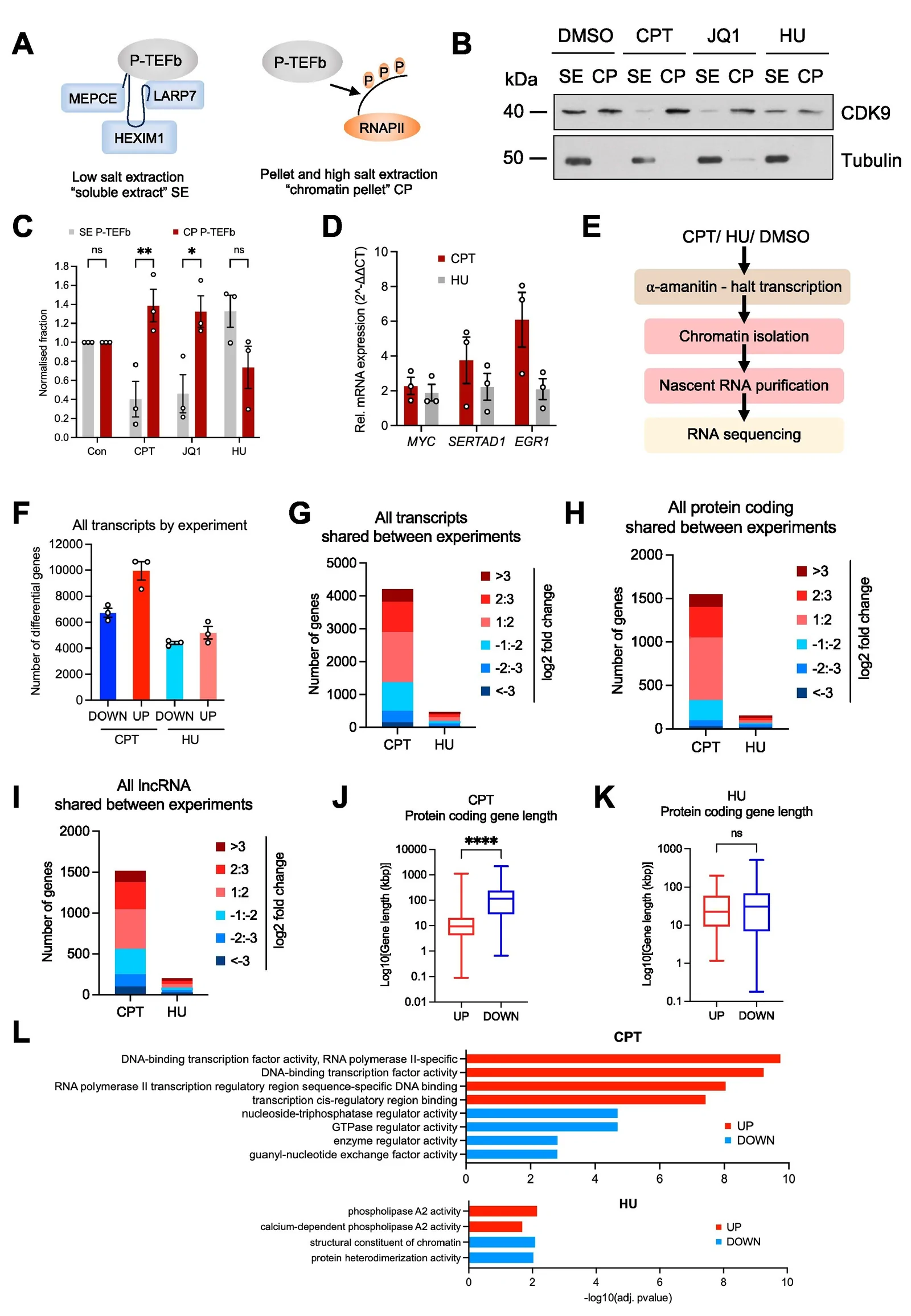
CPT, but not HU, activates a transcription stress response. (**A**) Principle of detecting soluble and chromatin-bound P-TEFb by cell fractionation. (**B**) Protein levels of CDK9 and Tubulin (fractionation control) in soluble extract (SE) and CP fractions after 2-h treatment with 10 mM CPT, 1 mM BET inhibitor (JQ1), or 200 mM HU. (**C**) CDK9 in SE (7SK-snRNP-bound) and NP (chromatin-bound) P-TEFb calculated as fraction of total CDK9 for each treatment and normalized to untreated controls. *n* = 3. (**D**) RT-qPCR analysis of *MYC, SERTAD1*, and *EGR1* expression after 2-h treatment with 10 mM CPT or 200 mM HU. *n* = 3. (**E**) Principle of chromatin RNA-seq approach. (**F**) Numbers of nascent transcripts significantly up- or downregulated after 2-h 10 mM CPT or 200 mM HU treatment in each repeat (log_2_-fold change). (**G**) Numbers of up- or downregulated transcripts shared between all repeats. (**H**) Numbers of up- or downregulated protein-coding transcripts shared between all repeats. (**I**) Numbers of up- or downregulated lncRNA transcripts shared between all repeats. (**J**) Gene lengths of shared transcripts up- or downregulated in response to CPT. (**K**) Gene lengths of shared transcripts up- or downregulated in response to HU. (**L**) Gene Ontology (molecular function) of genes significantly up- or downregulated after CPT or HU treatment. Column graphs show mean ± SEM. Two-way ANOVA or Mann–Whitney test, *P ≤ .05; **P ≤ .01; ^****^P ≤ .0001; ns: not significant.

To investigate the impact of CPT and HU on transcription stress, we used chromatin RNA-seq to quantify nascent transcripts [45]. Chromatin-bound nascent RNA was isolated 2 h after CPT or HU treatment and subjected to next-generation sequencing (Fig. 3E and Supplementary Fig. S2B–E). Data from three biological repeats showed that both CPT and HU treatments altered the synthesis of large numbers of transcripts in every experiment. CPT affected more transcripts than HU, with more transcripts up-regulated than down-regulated (Fig. 3F). When we considered only transcripts that were significantly altered across all three biological repeats, this revealed that only CPT induced a specific transcriptional response (Fig. 3G). In addition to protein-coding transcripts, transcripts consistently altered in response to CPT included non-coding RNAs such as lncRNAs, microRNAs, and snRNAs (Fig. 3H and I; Supplementary Table S1). Nascent RNA-seq had previously shown that both CPT and 4-NQO lead to a rapid (<1 h) relative decrease in longer transcripts and relative increase in shorter transcripts [8, 46]. Transcripts up-regulated in response to CPT were also shorter than down-regulated transcripts, consistent with transcription stress (Fig. 3J). In contrast, the transcripts consistently up- or downregulated in HU treatment were of similar length (Fig. 3K). Protein-coding genes up-regulated in response to CPT were enriched in genes involved in the regulation of RNAPII transcription and nucleic acid binding (Fig. 3L). These included genes that were previously found up-regulated in response to short (≤ 2 h) treatments with CPT, JQ1, or 4-NQO [8, 46, 47], such as the transcription factors *JUN, FOS, EGR1*, and *SERTAD1, HEXIM1* itself, and ribosomal protein genes. Comparatively fewer gene ontologies were enriched for genes down-regulated by CPT or altered in response to HU. Cell-cycle or DNA-damage response genes were largely unaffected by either treatment (Supplementary Table S1). Overall, these data are consistent with CPT, but not HU, inducing the P-TEFb relocation and transcriptional changes associated with a transcription stress response. This provides us with tools to investigate the roles of HEXIM1 and LARP7 in presence and absence of overt transcription stress.

### Limited evidence for HEXIM1 promoting transcription–replication conflicts

We speculated that CPT-induced release of P-TEFb might promote conflicts between transcription and replication, as previously suggested for JQ1 [19]. We first tested whether HEXIM1 or LARP7 depletion would prevent the relocalization of P-TEFb to chromatin. HEXIM1 and LARP7 depletion attenuated the CPT-induced increase in chromatin-bound P-TEFb (Fig. 4A–D; Supplementary Fig. S3A–D). However, especially in the LARP7 depletion experiments, a significant fraction of CDK9 could still relocate to chromatin (Fig. 4B and D; Supplementary Fig. S3C and D).

**Figure 4.**
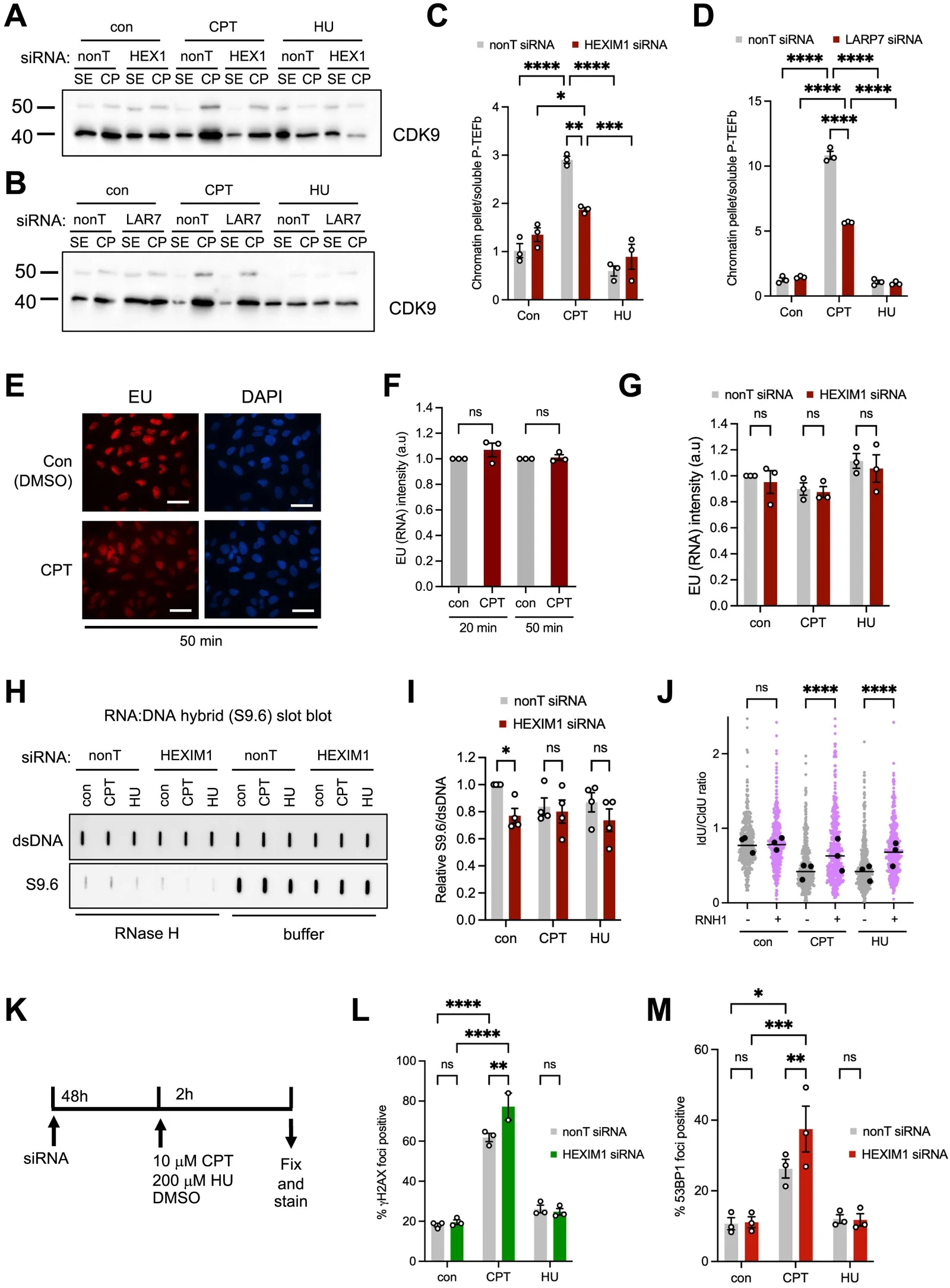
7SK-snRNP complex components do not promote transcription–replication conflicts. (**A**) Protein levels of CDK9 in SE and CP fractions after 2-h treatment with 10 mM CPT or 200 mM HU ± HEXIM1 siRNA (48 h). (**B**) Protein levels of CDK9 in SE and CP fractions ± LARP7 siRNA as in panel A. (**C**) Relative levels of SE (7SK-snRNP bound) versus NP (chromatin-bound) P-TEFb after treatment ± HEXIM1 siRNA as in panel (A). *n* = 3. (**D**) Relative levels of SE versus NP P-TEFb after treatment ± LARP7 siRNA as in panel (B). *n* = 3. (**E**) Representative images of nascent RNA labelling with 5-ethynyluridine (EU). DNA was detected using DAPI. Bars: 50 mm. (**F**) Nuclear EU intensities after treatment with 10 mM CPT and EU for 20 or 50 min. *n* = 3. (**G**) Nuclear EU intensities after treatment with 10 mM CPT or 200 mM HU (2 h) ± HEXIM1 siRNA. *n* = 3. (**H**) Slot blots of genomic DNA stained with S9.6 antibody (RNA:DNA hybrids) and dsDNA (loading control) after treatment with 10 mM CPT or 200 mM HU (20 min) ± HEXIM1 siRNA. RNase H treatment was used as control. (**I**) RNA:DNA hybrid quantification as in panel (H). *n* = 4. (**J**) IdU/CldU ratios after CPT or HU treatment overexpression of turboGFP-RNase H1 (+) or eGFP vector control (−) for 24 h. Data from three repeats. (**K**) Approach for siRNA depletion and 10 mM CPT or 200 mM HU treatment prior to immunostaining. (**L**) Percentages of cells with >10 gH2AX foci after 2-h CPT or HU ± HEXIM1 siRNA. *n* = 2–3. (**M**) Percentages of cells with >9 53BP1 foci after 2-h CPT or HU ± HEXIM1 siRNA. *n* = 3. Scatter blots show aggregates and medians (black points) of independent repeats with overall median (line). ANOVA or Kruskal–Wallis with multiple comparisons test, *P ≤ .05; **P ≤ .01; ***P ≤ .001; ^****^P ≤ .0001; ns: not significant.

We used EU incorporation assays to test whether P-TEFb release supports increased global nascent RNA synthesis after drug treatment, which could then promote conflicts between transcription and replication. However, CPT treatment did not increase global levels of nascent RNA synthesis (Fig. 4E and F), and HEXIM1 depletion did not further decrease global nascent RNA synthesis in cells treated with either CPT or HU (Fig. 4G). This is consistent with a previous report showing limited impact of 7SK loss on global transcription after UV irradiation [7]. Next, we tested whether HEXIM1 might be required for RNA:DNA hybrid formation in response to CPT treatment. A slot blot of isolated genomic DNA using the S9.6 antibody that detects RNA:DNA hybrids [48] showed that HEXIM1 depletion caused a small reduction in RNA:DNA hybrid levels in untreated cells, but there was no difference when combined with CPT or HU treatment for 20 min (Fig. 4H and I). We then transiently overexpressed ectopic human ribonuclease H1 (RNase H1) to reduce RNA:DNA hybrid levels. Because RNase H1 overexpression on its own slightly reduced replication fork speeds, we display IdU/CldU ratios for this experiment, and for other treatments that reduced fork speeds on their own. RNase H1 overexpression partially rescued replication fork slowing induced both by CPT and by HU (Fig. 4J and Supplementary Fig. S3E). This suggested that RNA:DNA hybrids are involved in replication fork slowing induced by both treatments, and not specific to CPT.

If HEXIM1 was responsible for promoting transcription–replication conflicts and thereby the source of replication stress, the HEXIM1 depletion might be expected to reduce the amount of DNA damage caused by CPT. Instead, HEXIM1 depletion slightly increased CPT-induced DNA damage as measured by gH2AX and 53BP1 nuclear foci formation (Fig. 4K–M). Taken together, these data did not support that HEXIM1-induced replication fork slowing is due to HEXIM1 promoting conflicts between transcription and replication.

### HEXIM1 and LARP7 are required for replication fork reversal and HR repair

We had shown that CPT and HU had different impacts on transcription and activation of transcription stress responses, but these drugs also differ further in the mechanisms by which they cause replication fork slowing. Specifically, fork slowing induced by CPT, but not by HU, depends on RAD51-mediated replication fork reversal [49] (Fig. 5A). We therefore asked whether the requirement for 7SK-snRNP components in CPT-induced fork slowing might be due to a role for the 7SK-snRNP in promoting fork reversal. We first confirmed the specific requirement for fork reversal in CPT-induced fork slowing by depleting fork reversal factor ZRANB3 (Fig. 5A and B). ZRANB3 depletion rescued replication fork slowing induced by CPT, but not by HU (Fig. 5C). We next depleted fork reversal and HR factor RAD51 (Fig. 5D). RAD51 depletion rescued CPT-induced fork slowing, and combination of RAD51 and HEXIM1 depletion had no additional effect on fork speeds, supporting that RAD51 and HEXIM1 may act in the same pathway (Fig. 5E).

**Figure 5.**
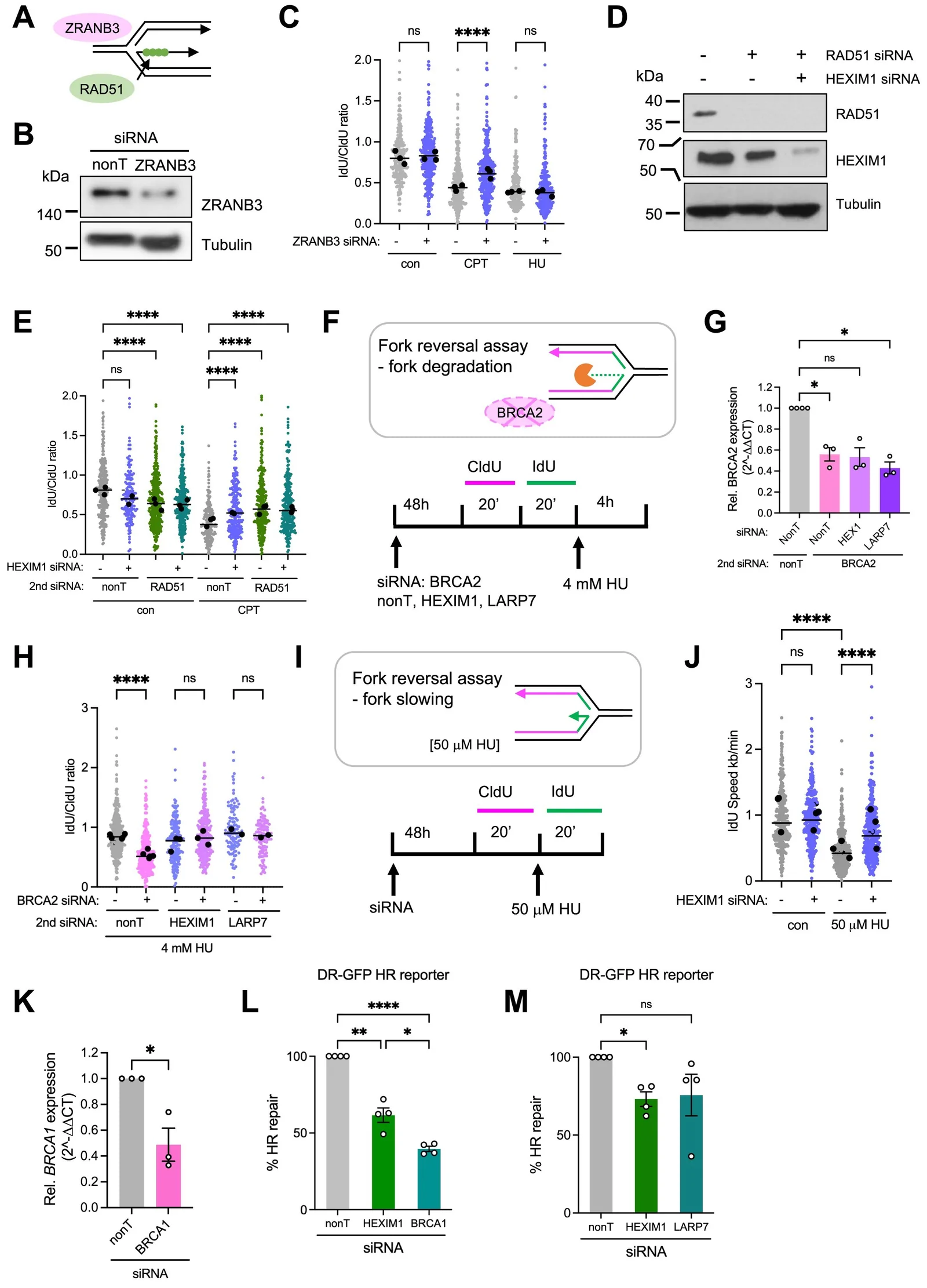
7SK-snRNP components are required for replication fork reversal and HR repair. (**A**) RAD51 and ZRANB3 promote replication fork reversal. (**B**) Protein levels of ZRANB3 and Tubulin (loading control) in U2OS cells 48 h after siRNA transfection. (**C**) IdU/CldU ratios after CPT or HU treatment in presence of ZRANB3 (+) or nonT (−) siRNA. Data from three repeats. (**D**) Protein levels of RAD51, HEXIM1, and Tubulin (loading control) in U2OS cells in presence of HEXIM1 (+) or nonT (−) siRNA ± RAD51 siRNA. (**E**) IdU/CldU ratios after CPT treatment ± HEXIM1 and/or RAD51 siRNA. Data from three repeats. (**F**) Approach for replication fork reversal assay based on fork degradation, using BRCA2 and/or HEXIM1 or LARP7 siRNA depletion prior to DNA fibre labelling followed by 4 mM HU (4 h). (**G**) RT-qPCR of BRCA2 messenger RNA (mRNA) expression in U2OS cells 48 h after nonT or BRCA2 siRNA ± HEXIM1 or LARP7 siRNA transfection. *n* = 3. (**H**) IdU/CldU ratios after 4 mM HU treatment ± BRCA2 and/or HEXIM1 or LARP7 siRNA. Data from five repeats (nonT), three repeats (HEXIM1), or two repeats (LARP7). (**I**) Approach for measuring fork reversal assay based on replication fork slowing in presence of 50 mM HU. (**J**) Replication fork speeds (IdU label) after 50 mM HU treatment in presence of HEXIM1 (+) or nonT (−) siRNA. Data from three repeats. (**K**) RT-qPCR of BRCA1 mRNA expression in U2OS cells 48 h after siRNA transfection. *n* = 3. (**L**) Relative HR repair by gene conversion in U2OS cells harbouring a DR-GFP reporter plasmid, ± HEXIM1 or BRCA1 siRNA. *n* = 4. (**M**) Relative HR repair by gene conversion in U2OS cells harbouring a DR-GFP reporter plasmid, ± HEXIM1 or LARP7 siRNA. *n* = 4. Scatter blots show aggregates and medians (black points) of independent repeats with overall median (line). ANOVA or Kruskal–Wallis with multiple comparisons test, *P ≤ .05; **P ≤ .01; ^****^P ≤ .0001; ns: not significant.

To investigate a role for HEXIM1 in replication fork reversal more directly, we employed a DNA fibre labelling protocol that can measure fork reversal capacity. In absence of BRCA2, reversed forks are vulnerable to nuclease degradation. This leads to shortening of the nascent labelled DNA strand during a prolonged replication block with a high dose of HU. If nascent strands are not shortened under these conditions, this is indicative of a defect in fork reversal [50] (Fig. 5F). U2OS cells were treated with siRNA against BRCA2, HEXIM1, or LARP7 alone or in combination (Fig. 5G and Supplementary Fig. S4A), and DNA fibre labelling with CldU and IdU was performed as before, but the IdU label was followed by a 4-h block with 4 mM HU. BRCA2 depletion alone led to IdU track shortening, as indicated by a decreased IdU/CldU length ratio, but when HEXIM1 or LARP7 was depleted, IdU tracks did not shorten either with or without BRCA2 depletion (Fig. 5H). This suggests that HEXIM1 is required for replication fork reversal, potentially through a role in RAD51 function. As an alternative approach to investigating the role of HEXIM1 in fork reversal, we treated cells with 50 mM HU, where fork slowing is mostly caused by transcription-dependent fork reversal rather than nucleotide depletion [28]. HEXIM1 depletion rescued replication fork slowing induced by 50 mM HU, suggesting that HEXIM1 is required for fork reversal even when there is no transcription stress (Fig. 5I and J).

If fork reversal is prevented, then specialized DNA polymerases such as PRIMPOL or REV1 may take over and support higher replication fork speeds [51]. However, neither PRIMPOL nor REV1 appeared to be required for the increased replication fork progression in presence of CPT and absence of HEXIM1 (Supplementary Fig. S4B–D).

Since RAD51 is also required for HR repair, we tested whether HEXIM1 and LARP7 promote HR repair using a U2OS cell line harbouring the DR-GFP reporter construct [52]. After the rare-cutting I-SceI endonuclease induces a double-ended DSB (deDSB) in the reporter, expression of WT GFP shows HR repair of this site by gene conversion. HEXIM1 depletion strongly reduced HR repair in this assay, compared to BRCA1 depletion used as a positive control (Fig. 5K and L; Supplementary Fig. S4E). This supports that HEXIM1 promotes HR repair independently of endogenous sources of replication stress, such as conflicts between transcription and replication. Two previous studies reported that LARP7 loss either increased or slightly decreased HR in the DR-GFP reporter construct [20, 22], potentially suggesting a separate role for LARP7 compared to other 7SK-snRNP components such as MEPCE [22] and HEXIM1. We therefore again probed the involvement of LARP7 in HR at direct or deDSBs. In our hands, LARP7 depletion overall decreased HR in the DR-GFP reporter assay, albeit to a slightly lesser extent than HEXIM1 depletion (Fig. 5M). HEXIM1 or LARP7 depletion did not alter cell cycle distribution (Supplementary Fig. S4F). These data support that, like MEPCE, HEXIM1 and LARP7 promotes HR, although LARP7 may be less able to promote HR repair at deDSBs.

### HEXIM1 and LARP7 promote RAD51 recruitment in response to a subset of DNA-damaging agents

We next used a range of DNA-damaging treatments to further investigate the role of 7SK-snRNP components in HR activation in presence of different levels of transcription stress. Nuclear RAD51 foci formation was used as a measure of RAD51 recruitment to damage sites and initiation of HR (Fig. 6A and Supplementary Fig. S5A). Treatment with either 10 mM CPT or 200 mM HU for 2 h induced similar levels of RAD51 foci. Both HEXIM1 or LARP7 depletion reduced RAD51 foci induction by CPT, which causes transcription stress, but did not reduce RAD51 foci in response to HU, which had not caused transcription stress (Fig. 6B and C). Similar results were observed in other human cancer cell lines, HeLa and HCT116 (Supplementary Fig. S5B and C), and RAD51 foci formation in response to CPT was decreased in HAP1 LARP7 or 7SK KO cells with or without HEXIM1 co-depletion (Fig. 6D). Short-term DRB treatment to inhibit P-TEFb prevented CPT-, but not HU-induced RAD51 foci formation, and combining DRB treatment with HEXIM1 depletion did not cause a further reduction in foci (Fig. 6E). WT HEXIM1, but not the PDND mutant, restored CPT-induced RAD51 formation (Fig. 6F). This suggests that RAD51 recruitment in response to 200 mM HU, which does not cause transcription stress, requires neither 7SK-snRNP components nor CDK9 activity or RNAPII elongation.

**Figure 6.**
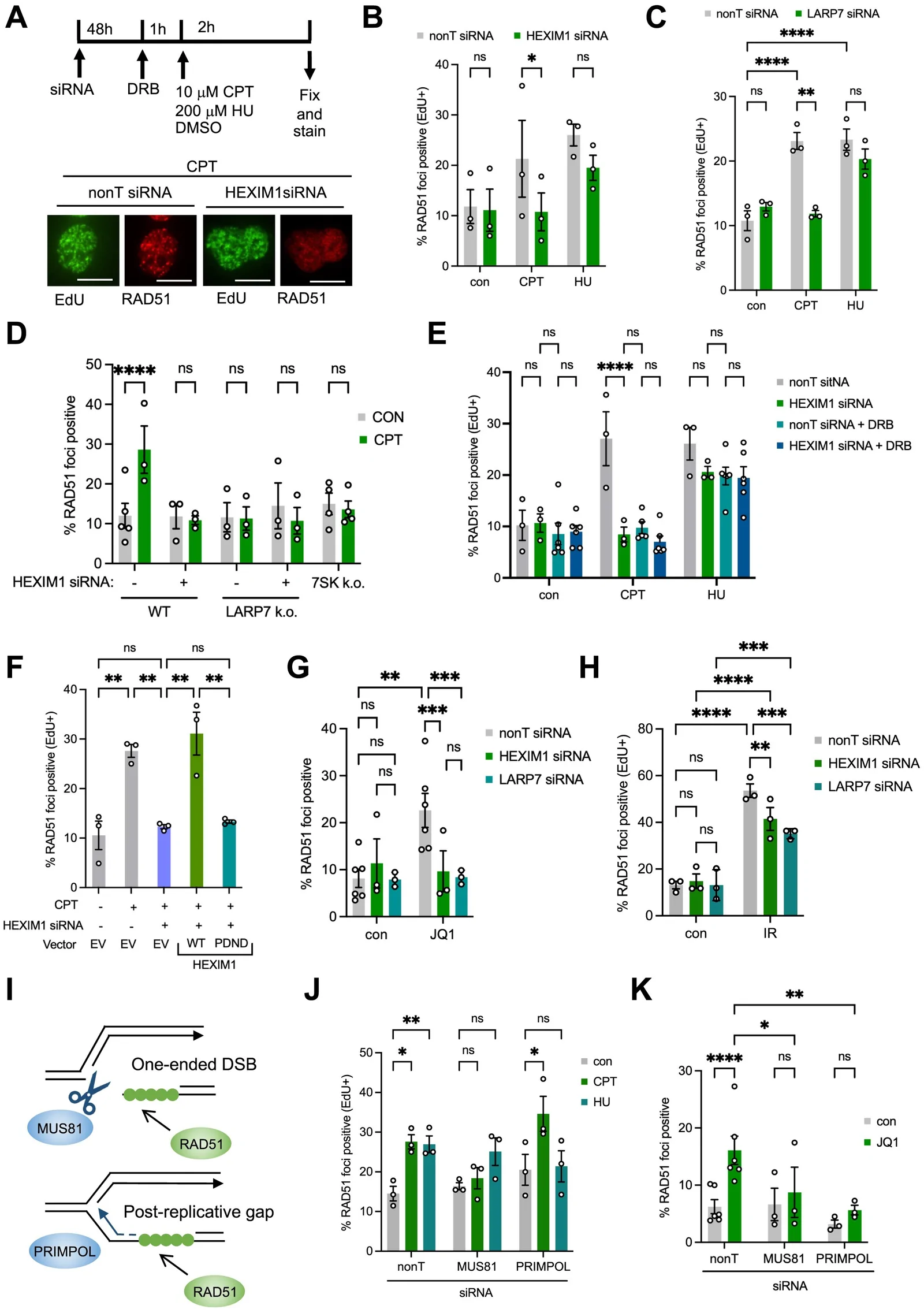
7SK-snRNP components are required for RAD51 recruitment in response to agents that cause transcription stress. (**A**) Approach for siRNA depletion or small molecule inhibitor treatment prior to 2-h treatment with 10 mM CPT or 200 mM HU, followed by immunostaining. Bottom: Representative images of RAD51 foci in U2OS cells after 2-h treatment with 10 mM CPT ± HEXIM1 siRNA. Bars: 10 mm. (**B**) Percentages of EdU-positive cells with >5 RAD51 foci after CPT or HU treatment ± HEXIM1 siRNA. *n* = 3. (**C**) Percentages of EdU-positive cells with >5 RAD51 foci after CPT or HU treatment ± LARP7 siRNA. *n* = 3. (**D**) Percentages of EdU-positive cells with >5 RAD51 foci after CPT treatment ± HEXIM1 siRNA in WT, LARP7 k.o., or 7SK k.o. HAP1 cells. *n* = 3 (7SK k.o.: *n* = 4; WT nonT: *n* = 5). (**E**) Percentages of EdU-positive cells with >5 RAD51 foci after CPT or HU treatment ± DRB in presence of nonT or HEXIM1 siRNA (*n* = 3–6). (**F**) Percentages of EdU-positive cells with >5 RAD51 foci after CPT treatment in presence of nonT (−) or HEXIM1 (+) siRNA and transient transfection with EV or plasmids encoding WT or PDND HEXIM1. *n* = 3. (**G**) Percentages of cells with >5 RAD51 foci after 1 mM JQ1 treatment ± HEXIM1 or LARP7 siRNA. *n* = 3 (nonT *n* = 6). (**H**) Percentages of EdU-positive cells with >5 RAD51 foci 4 h after 2 Gy X-ray irradiation ± HEXIM1 or LARP7 siRNA. *n* = 3. (**I**) MUS81 and PRIMPOL generate one-ended DSBs and post-replicative gaps, substrates for RAD51 loading. (**J**) Percentages of EdU-positive cells with >5 RAD51 foci after CPT or HU treatment ± MUS81 or PRIMPOL siRNA. *n* = 3. (**K**) Percentages of cells with >5 RAD51 foci after 1 mM JQ1 treatment (4 h) ± MUS81 or PRIMPOL siRNA. *n* = 3 (nonT *n* = 6). Column graphs show mean ± SEM. ANOVA with multiple comparisons test, *P ≤ .05; **P ≤ .01; ***P ≤ .001; ^****^P ≤ .0001; ns: not significant.

HEXIM1 and LARP7 depletion also reduced RAD51 foci formation in response to JQ1, another agent that causes transcription stress (Fig. 6G). It was previously reported that LARP7 loss increased RAD51 foci formation after X-ray irradiation [20]. In our hands, both LARP7 and HEXIM1 depletion slightly decreased X-ray induced RAD51 foci formation (Fig. 6H). Similarly, MEPCE depletion has been reported to decrease RAD51 recruitment at a subset of endonuclease-induced deDSBs [22].

We next investigated whether different lesion types underlie the differential requirements for RAD51 foci formation in response to CPT and JQ1 versus HU, as RAD51 can form foci either at DSBs or at post-replicative single-stranded gaps [15]. We depleted the structure-specific endonuclease MUS81 to prevent seDSB formation at replication forks [53] and depleted PRIMPOL to prevent post-replicative gap formation (Fig. 6I and Supplementary Fig. S4B and S5D). This showed that CPT-induced RAD51 foci formed at MUS81-induced seDSBs as expected, while HU-induced RAD51 foci appeared to form at PRIMPOL-induced post-replicative gaps (Fig. 6J). JQ1-induced RAD51 foci depended on both MUS81 and PRIMPOL (Fig. 6K). It is therefore possible that 7SK-snRNP promotes RAD51 foci formation specifically at collapsed replication forks rather than post-replicative gaps.

Since HEXIM1 and LARP7 are involved in transcriptional regulation, we used western blot or RT-qPCR to verify that their depletion did not affect expression of RAD51, ZRANB3, MUS81, PRIMPOL, BRCA1, and BRCA2. LARP7 depletion slightly reduced PRIMPOL and BRCA1 mRNA expression (Supplementary Fig. S5E and F), but there were no changes in the levels of any of these factors under any other condition (Supplementary Fig. S5E–H).

Taken together, these data suggested that across a panel of treatments that induce DNA lesions that activate HR, the 7SK-snRNP components promote RAD51 recruitment in response to those treatments that cause transcription stress.

### LARP7 promotes RAD51 foci formation and replication fork slowing separately from DNA end resection and BRCA1

We next investigated potential mechanisms that could explain the RAD51 loading defect in absence of 7SK-snRNP components. First, an inability to recruit RAD51 to sites of DNA damage can result from defects in DNA end resection, which can be assessed through levels of phospho-serine4/8 RPA after 1-h CPT treatment [54] (Fig. 7A). HEXIM1 or LARP7 depletion did not reduce phospho-serine4/8 RPA formation in response to CPT (Fig. 7B and C; Supplementary Fig. S6A). This suggested a RAD51 recruitment defect rather than a DNA end resection defect.

**Figure 7.**
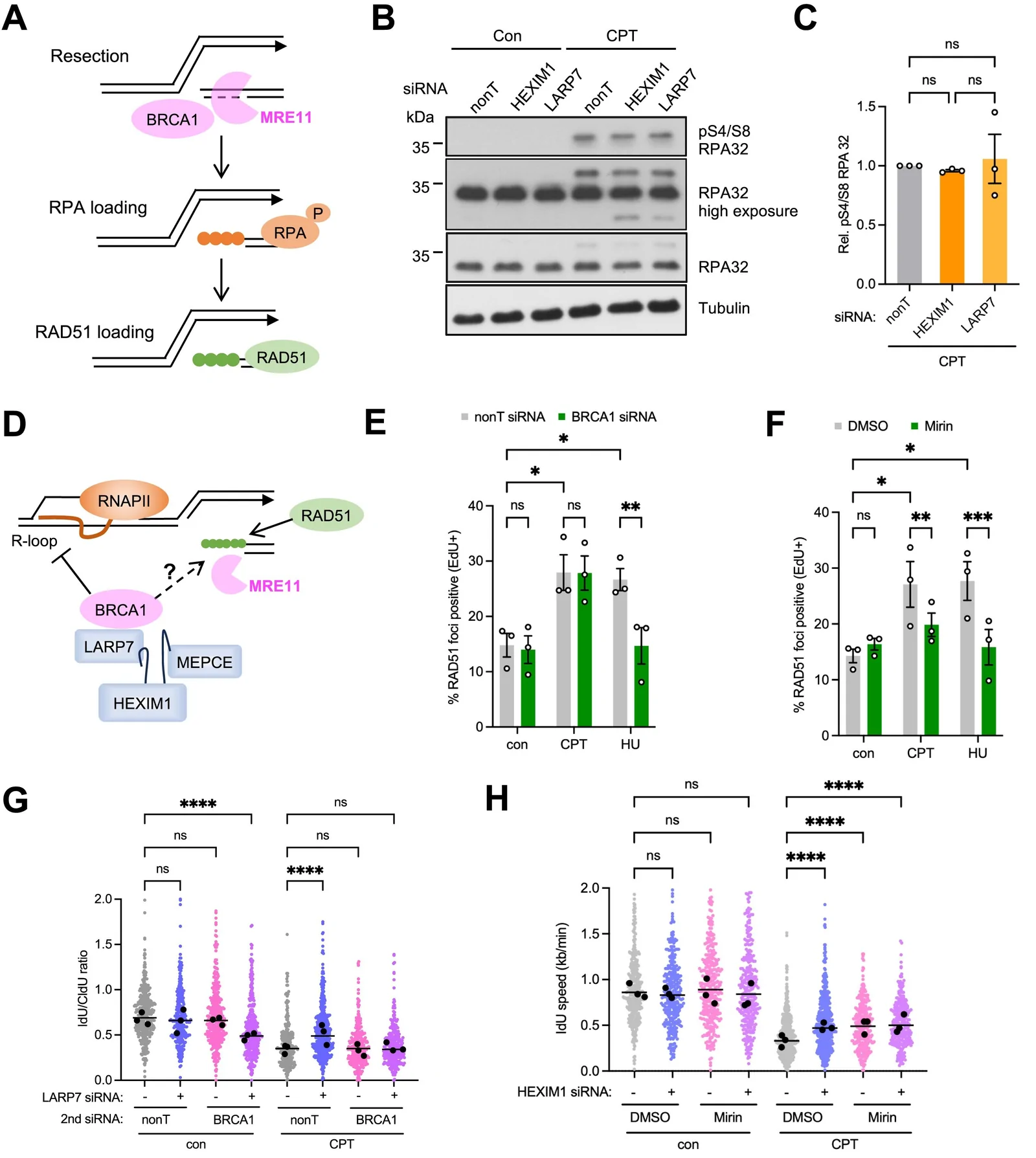
LARP7 and HEXIM1 promote RAD51 recruitment and fork reversal independently of DNA end resection and BRCA1. (**A**) Roles of BRCA1, MRE11, and phospho-RPA in DNA end resection and RAD51 loading. (**B**) Protein levels of phospho-serine 4/8 RPA32, RPA32 (loading control), and Tubulin (loading control) after 2-h 10 mM CPT treatment ± HEXIM1 or LARP7 siRNA. (**C**) Quantified levels of phospho-serine 4/8 RPA normalized to RPA32 after treatment as in B, relative to nonT siRNA. *n* = 3. (**D**) Interaction between LARP7 and BRCA1 suppresses R-loops and replication stress and might be involved in 7SK-snRNP-dependent RAD51 loading. (**E**) Percentages of EdU-positive cells with >5 RAD51 foci after CPT or HU treatment ± BRCA1 siRNA. *n* = 3. (**F**) Percentages of EdU-positive cells with >5 RAD51 foci after CPT or HU treatment ± MRE11 inhibitor (Mirin) treatment. *n* = 3. (**G**) IdU/CldU ratios after CPT treatment in presence of LARP7 (+) or nonT (−) siRNA ± BRCA1 siRNA. Data from three repeats. (**H**) IdU/CldU ratios after CPT treatment ± HEXIM1 siRNA and/or MRE11 inhibitor (Mirin) treatment. Data from three repeats. Column graphs show mean ± SEM. Scatter blots show aggregates and medians (black points) of independent repeats with overall median (line). ANOVA or Kruskal–Wallis with multiple comparisons test, *P ≤ .05; **P ≤ .01; ***P ≤ .001; ^****^P ≤ .0001; ns: not significant.

Second, LARP7 has been reported to physically interact with BRCA1, with BRCA1 promoting LARP7 degradation to facilitate HR [20, 22] (Fig. 7D). Intriguingly, we observed that CPT-induced RAD51 foci formation appeared to not require BRCA1 (Fig. 7E), although it required the activity of MRE11, which is essential for DNA end resection (Fig. 7F). This agrees with a recent report that end resection at seDSBs such as those induced by CPT does not require BRCA1, with *BRCA1*^−/−^ cells displaying residual CPT-induced RAD51 foci [55]. We then combined siRNA depletion of BRCA1 and LARP7 to investigate whether LARP7 might interact with BRCA1 to promote CPT-induced fork slowing (Supplementary Fig. S6B and C). As reported before [21], combined loss of BRCA1 with LARP7 caused severe replication stress in unchallenged cells (Supplementary Fig. S6D). However, BRCA1 was not required for CPT-induced fork slowing, indicating that it does not act in the same pathway as LARP7 (Fig. 7G). Again, CPT-induced fork slowing required MRE11 activity (Fig. 7H). This raises the possibility that LARP7 can promote RAD51 functions that are independent of BRCA1.

Because ongoing transcription favours HR repair [18, 56] and helps recruit HR factors [57], we tested whether HEXIM1 or LARP7 depletion altered transcription of the eGFP gene after break induction. We also tested levels of antisense transcription, which could be indicative of *de novo* RNA synthesis at the 3′-overhang generated by resection [58, 59] (Supplementary Fig. S7A). In our hands, antisense transcription was not reliably increased after DSB induction, and this increase was not dependent on CtIP (resection), HEXIM1, or LARP7 (Supplementary Fig. S7B). While HEXIM1 or LARP7 depletion slightly altered the basal expression of eGFP mRNA, HEXIM1 or LARP7 were not required for eGFP mRNA transcription after DSB induction (Supplementary Fig. S7C and D). HEXIM1 or LARP7 depletion did also not prevent the rapid induction of transcription factors *EGR1* and *SERTAD1* in response to CPT (Supplementary Fig. S7E).

### HEXIM1 and LARP7 promote cell survival in response to CPT and HU, but not olaparib

We had previously shown that HEXIM1 depletion sensitizes U2OS cells to JQ1 [19]. To probe the effect of HEXIM1 or LARP7 loss on cell survival of replication stress-inducing and DNA-damaging treatments, we performed colony formation assays in the presence of CPT or HU, as well as PARP inhibitor olaparib. Both HEXIM1 and LARP7 depletion did not reduce plating efficiency of untreated cells (Fig. 8A), but sensitized U2OS cells to CPT (Fig. 8B) and to a lesser extent to HU (Fig. 8C). However, loss of HEXIM1 or LARP7 had a relatively minor impact on sensitivity to olaparib, unlike what would be expected for canonical HR defects (Fig. 8D).

**Figure 8.**
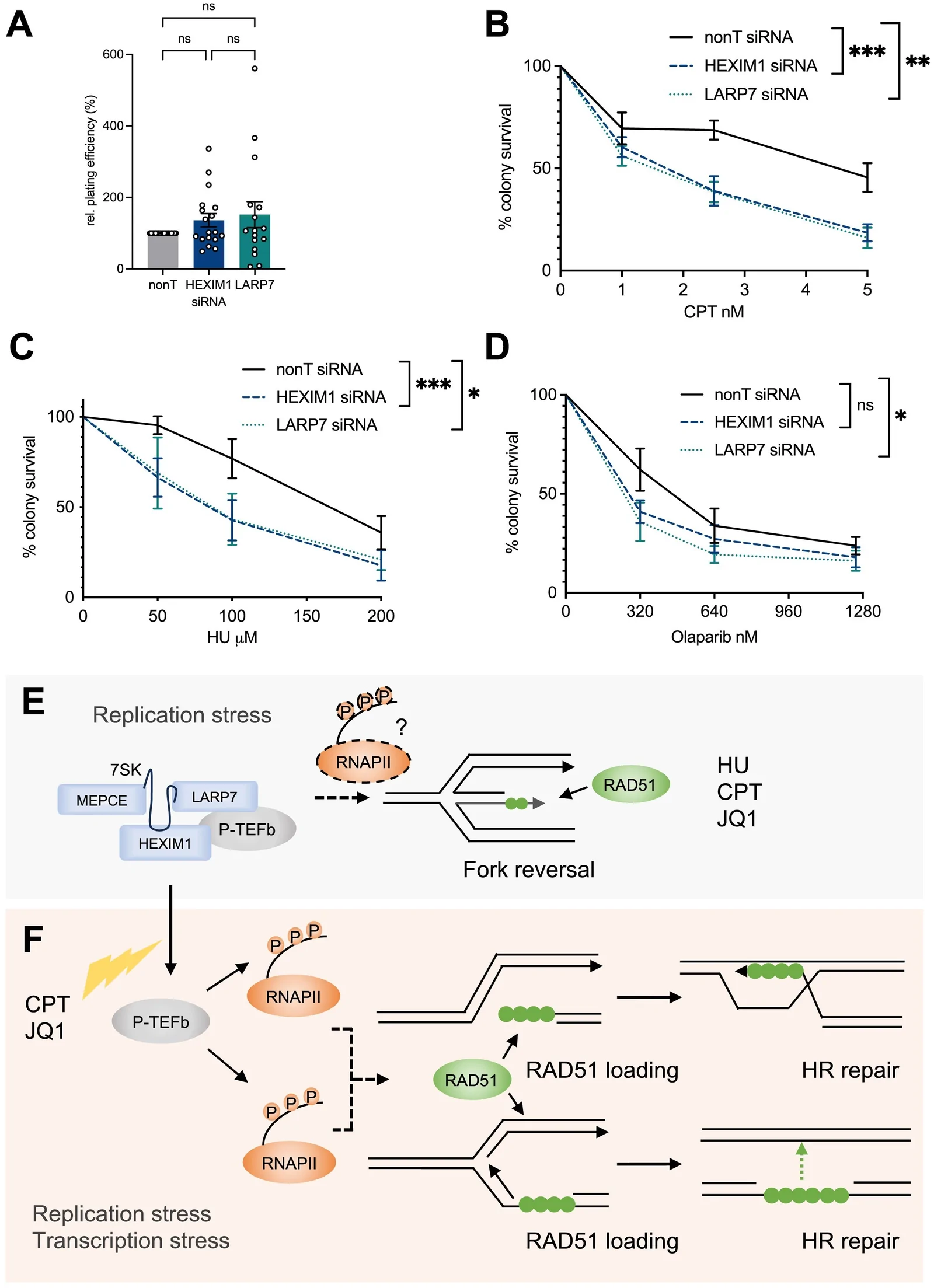
The 7SK-snRNP promotes survival in response to replication stress. (**A**) Percentage plating efficiency of U2OS cells treated with HEXIM1 or LARP7 siRNA in colony assays, relative to nonT control siRNA. *n* = 18 (HEXIM1 siRNA); *n* = 16 (LARP7 siRNA). (**B**) Colony survival assay of U2OS cells after continuous treatment with CPT at the indicated concentrations ± HEXIM1 or LARP7 siRNA. *n* = 4–5. (**C**) Colony survival assay of U2OS cells after continuous treatment with HU at the indicated concentrations ± HEXIM1 or LARP7 siRNA. *n* = 4–7. (**D**) Colony survival assay of U2OS cells after continuous treatment with PARP inhibitor (olaparib) at the indicated concentrations ± HEXIM or LARP7 siRNA. *n* = 4. (**E**) 7SK-snRNP complex components are required for replication fork reversal under all replication stress conditions, acting in the same pathway as RAD51. (**F**) 7SK-snRNP complex components are required for RAD51 recruitment and HR repair at DSBs and post-replicative gaps specifically under conditions of transcription stress. These two scenarios could reflect separate functions of the 7SK-snRNP, or differential requirements for RNAPII activity regulation at replication forks versus DNA damage sites. ANOVA with multiple comparisons test, *P ≤ .05; **P ≤ .01; ***P ≤ .001; ns: not significant.

Taken together, this supports that 7SK-snRNP components support HR and survival in response to a subset of treatments, and notably treatments that induce transcription stress.

## Discussion

We have shown that components of the 7SK-snRNP promote replication fork reversal, HR repair, and cell survival in response to DNA-damaging agents. This role becomes especially important under treatment conditions where there is evidence of transcription stress and transcriptional rewiring. 7SK-snRNP could promote fork reversal and HR through the same pathway, but it might also act through two different pathways, especially since 7SK-snRNP seems to promote fork reversal under a wider range of treatment conditions (Fig. 8E and F).

LARP7 and MEPCE are RNA end protection factors required for 7SK RNA stability, so that LARP7 loss, like 7SK RNA loss, disrupts the entire complex [60]. In contrast, HEXIM1 is the essential regulator of the P-TEFb interaction, so that its loss would preserve the rest of the complex but abrogate its ability to control P-TEFb activity [9]. *RN7SK* knockout cells are viable, suggesting that the complex is not required for normal transcription [7]. Nevertheless, acute 7SK-snRNP loss by RNAi depletion of LARP7 can result in a baseline shift of P-TEFb towards the active state [60], and both acute and chronic 7SK-snRNP loss prevent the DNA damage–induced release of additional P-TEFb [7].

A sizeable fraction of HEXIM1 exists outside of the 7SK-snRNP and in a ribonucleoprotein complex with NEAT1 and DNA-PK [7, 61], and HEXIM1 promotes p53 activation [62]. LARP7 interacts with BRCA1 [20], while MEPCE was recently suggested to recruit the histone chaperone FACT complex for R-loop resolution [22]. HEXIM1 or LARP7 depletion reduced but did not abolish CDK9 recruitment to chromatin in response to high doses of CPT (Fig. 4C and D). This could indicate that 7SK-snRNP is still partly functional after HEXIM1 or LARP7 depletion. However, the existence of additional, yet unknown, soluble P-TEFb complexes has been postulated [63], which could contribute to CDK9 recruitment in response to stress. Nevertheless, loss of HEXIM1, LARP7, or 7SK RNA has similar effects on replication stress phenotypes, and all 7SK-snRNP components promote RAD51 loading and HR activity in several conditions. Together with the results of using DRB and p38 inhibitors and a HEXIM1 mutant that cannot interact with P-TEFb, this is most consistent with a requirement for all three proteins in connection with 7SK-snRNP-mediated control of P-TEFb activity rather than for their independent roles outside the complex.

The reduced CPT- and JQ1-induced fork slowing after loss of HEXIM1, LARP7, or 7SK RNA is likely due to reduced fork reversal. Our data and data by others [22] support that all 7SK-snRNP components promote RAD51 recruitment to sites of DNA damage, which could include stressed replication forks. However, release of P-TEFb from the 7SK-snRNP appears to be only required for HR responses such as RAD51 foci formation and DSB repair, but not for fork reversal. While low-dose HU treatment does not induce signs of transcription stress, HU-induced fork reversal nevertheless depends on P-TEFb activity [28]. The 7SK-snRNP could promote RAD51 recruitment to challenged replication forks via its role in regulating transcription.

In contrast, 7SK-snRNP appears to promote HR functions such as RAD51 foci formation specifically under conditions of transcription stress. In this context, transcriptional repression observed at endonuclease-induced deDSBs [64] supports that DSBs in highly transcribed genes, such as in the DR-GFP reporter, could activate a transcription stress response. HU treatments at higher doses or for prolonged periods might also cause transcription stress: 3 mM HU stimulates CDK9 recruitment to chromatin [65]. However, our data might also support that 7SK-snRNP promotes RAD51 foci formation specifically at collapsed replication forks, or even at endonuclease-induced DSBs in general. This possibility will be subject to further investigation.

The 7SK-snRNP could be required for global transcription activity, which generally favours HR repair [18], or for HR gene expression. However, this is not consistent with the modest effects of 7SK-snRNP loss on global transcription levels after DNA damage or the fact that JQ1 treatment, while releasing P-TEFb from the 7SK-snRNP, nevertheless suppresses transcription of most protein-coding genes [47]. While published data show some reduced HR gene expression in cells lacking LARP7, this was not accompanied by major changes in BRCA1, BRCA2, or RAD51 protein levels [20].

Release of P-TEFb from the 7SK-snRNP could be required for gene transcription specifically in damaged regions. For example, ongoing transcription helps recruit HR factors to chromatin behind replication forks [57]. While transcription of protein-coding genes near endonuclease-induced deDSBs is rapidly suppressed [64], P-TEFb could potentially counteract this repression to promote HR. After loss of 7SK RNA or MEPCE, RNAPII accumulates in gene bodies away from the transcription start site [7, 21], and this could further prevent proper regulation of RNAPII pause release in response to DNA damage. We observed that HEXIM1 or LARP7 depletion both slightly altered the expression of mRNA from the DR-GFP reporter construct (Supplementary Fig. S6C and D), suggesting that deregulation of RNAPII activity could contribute to the defective HR repair of the construct.

Furthermore, recruitment of RNAPII followed by *de novo* RNA synthesis is proposed to promote HR repair at deDSBs [58, 59] and to generate RNA:DNA hybrids at stalled forks to aid fork protection [66]. *De novo* RNA synthesis at deDSBs is contested, and other studies support that RNA:DNA hybrids accumulate at deDSBs but are derived from pre-existing transcripts [56, 67]. MEPCE has been implicated in resolving these RNA:DNA hybrids at a subset of deDSBs by interacting with and recruiting hybrid-resolving factors such as FACT, although increased RNA:DNA hybrids at breaks could also be a consequence of defective repair [22]. In our hands, antisense transcription was not reliably induced after deDSB induction, even though we occasionally observed increased transcript levels after depletion of HEXIM1, LARP7, or CtIP. However, any *de novo* transcripts at breaks might be transiently produced and rapidly processed, preventing reliable detection by PCR-based methods.

Finally, control of P-TEFb through 7SK-snRNP could promote specific types of RNA synthesis that regulate DNA repair factors, even away from the sites of DNA damage [68]. Finally, CDK9 itself has been implicated in promoting HR through interaction with BRCA1–BARD1 [69] and in promoting replication stress responses through an interaction with ATR, although there was no direct evidence that CDK9 promotes ataxia telangiectasia and Rad3-related (ATR) signalling [65].

We previously suggested that transcription–replication conflicts play a role in 7SK-snRNP-mediated fork slowing, and our data cannot completely rule this out. Neither REV1 nor PRIMPOL is required for faster fork progression in absence of HEXIM1, which could support that there is not only no fork reversal but also no obstacle. However, 7SK-snRNP could help promote global fork reversal away from damage sites [70]. The differential effect of CDK9 inhibition on CPT- versus HU-induced RAD51 foci formation also suggests that transcription is involved in generating the lesions that require RAD51 for repair. However, this need not suggest transcription–replication conflicts because CPT causes genotoxic stress by interfering with the transcription machinery directly. JQ1 treatment does not cause any known sources of replication stress except increased global RNA synthesis and R-loop accumulation [19, 71, 72]. Nevertheless, JQ1 treatment only induced DNA damage when HEXIM1 was depleted, supporting that even here HEXIM1’s role is to promote RAD51 recruitment and DNA repair [19].

RNAPII activity promotes replication fork slowing, but rather than being due to transcription–replication conflicts, this could be a sign of a functional replication stress response. Future research should continue to investigate the relationship between transcription and replication stress responses. As combined loss of BRCA1 and either LARP7 or MEPCE has been reported to be synthetic lethal [21], and HEXIM1- or LARP7-depleted cells are hypersensitive to transcription stress-inducing treatments, this could have implications for the future development of targeted cancer therapies.

## Supplementary Material

gkag1021_Supplemental_Files

## Data Availability

This study did not generate unique new reagents and does not report original code. RNA sequencing data generated and analysed during this study are publicly available in the GEO repository under accession number GSE309660, https://www.ncbi.nlm.nih.gov/geo/query/acc.cgi?acc=GSE309660. UCSC Genome Browser tracks are available at https://genome.ucsc.edu/s/Kanhere/Bowry_ChrRNASeq.

## Notes

Present address: Division of Medical Sciences, Chester Medical School, University of Chester, Chester CH1 4BJ, United Kingdom
