# Supplementary material for: The 7SK small nuclear ribonucleoprotein links the cell responses to transcription and replication stress by promoting replication fork reversal and homologous recombination
Source: Nucleic Acids Res. 2026 Oct 29;54(20):gkag1021. doi: 10.1093/nar/gkag1021 (PMC13717190; doi:10.1093/nar/gkag1021)
Supplement: Supplementary file 1 [file gkag1021_supplemental_files.zip › Bowry et al Supplementary Data.pdf]

The 7SK small nuclear ribonucleoprotein links the cell responses to transcription and replication stress by promoting replication fork reversal and homologous recombination

Akhil Bowry, Claire Wilson, Richard D.W. Kelly, Rosanna J. Wilkins, Alexandra K. Walker, Christy S. Varghese, Zahraa A.M. Alshatti, Siobhan Murphy-Hollies, Joanna R. Morris, Sylvain Egloff, Aditi Kanhere, Eva Petermann

Supplementary Data

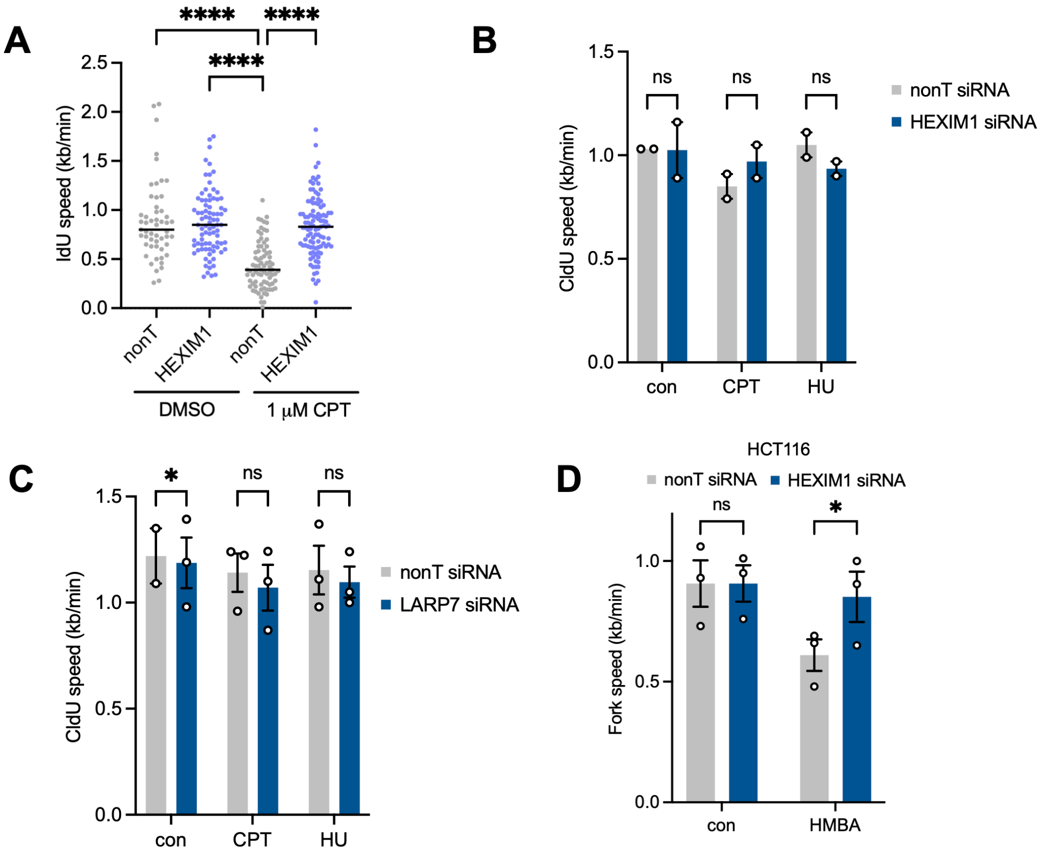

Figure S1. 7SK-snRP component depletion rescues replication fork slowing.
A) Second label (IdU) speeds in U2OS cells treated with non-targeting (nonT) or HEXIM1 siRNA for 48 h, followed by treatment with CldU for 20 min and IdU plus DMSO (con) or 1 µM CPT for 20 min. Data from 1 repeat. B) First label (CldU) speeds in U2OS cells treated with non-targeting (nonT) or HEXIM1 siRNA for 48 h, followed by treatment with CldU for 20 min and IdU plus DMSO (con), 10 µM CPT or 200 µM HU for 20 min. n=2. C) First label (CldU) speeds in U2OS cells treated with non-targeting (nonT) or LARP7 siRNA for 48 h, followed by treatment with CldU for 20 min and IdU plus DMSO (con), 10 µM CPT or 200 µM HU for 20 min. n=2-3. D) Replication fork speeds in HCT116 cells (CldU and IdU label) after HMBA treatment +/- HEXIM1 siRNA. n=3.
Scatter graphs show aggregate of independent repeat with median (line). Column graphs show mean ± SEM. ANOVA or Kruskal-Wallis with multiple comparisons test, * $p \leq 0.05$; **** $p \leq 0.0001$; ns: not significant.

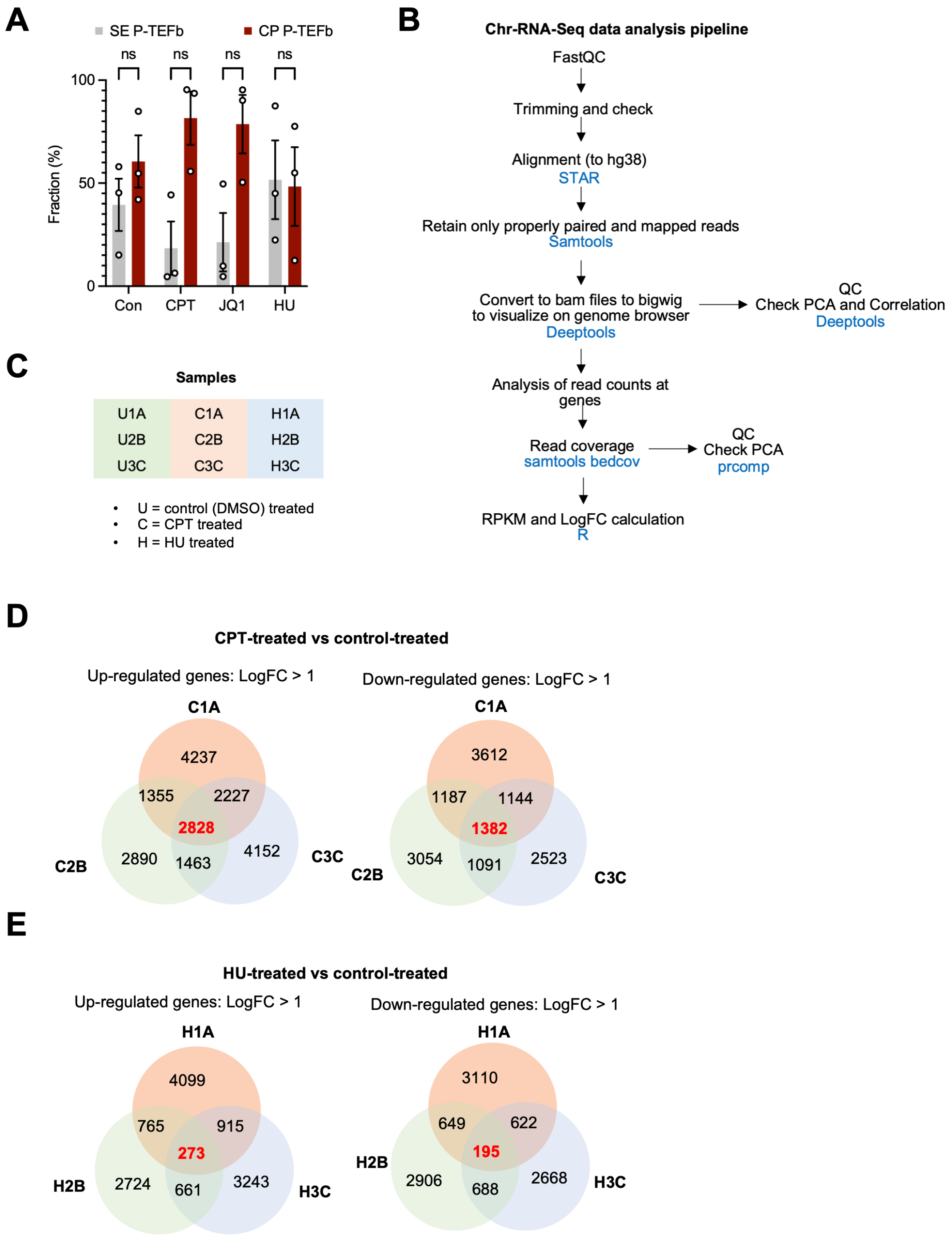

Figure S2. Impact of CPT and HU on the relocation of P-TEFb and nascent RNA synthesis.
A) Quantification of relative CDK9 band intensity in U2OS cells after 2 h treatment with 10 µM CPT, 200 µM HU or 1 µM JQ1. n=3. B) Workflow for computational analysis of chromatin RNA-seq. C) Schematic of treatment conditions used for chromatin RNA-seq. D) Venn diagrams of up-regulated and down-regulated in CPT- versus control-treated cells. E) Venn diagrams of up-regulated and down-regulated in HU- versus control-treated cells.
Column graphs show mean ± SEM. ANOVA with multiple comparisons test, ns: not significant.

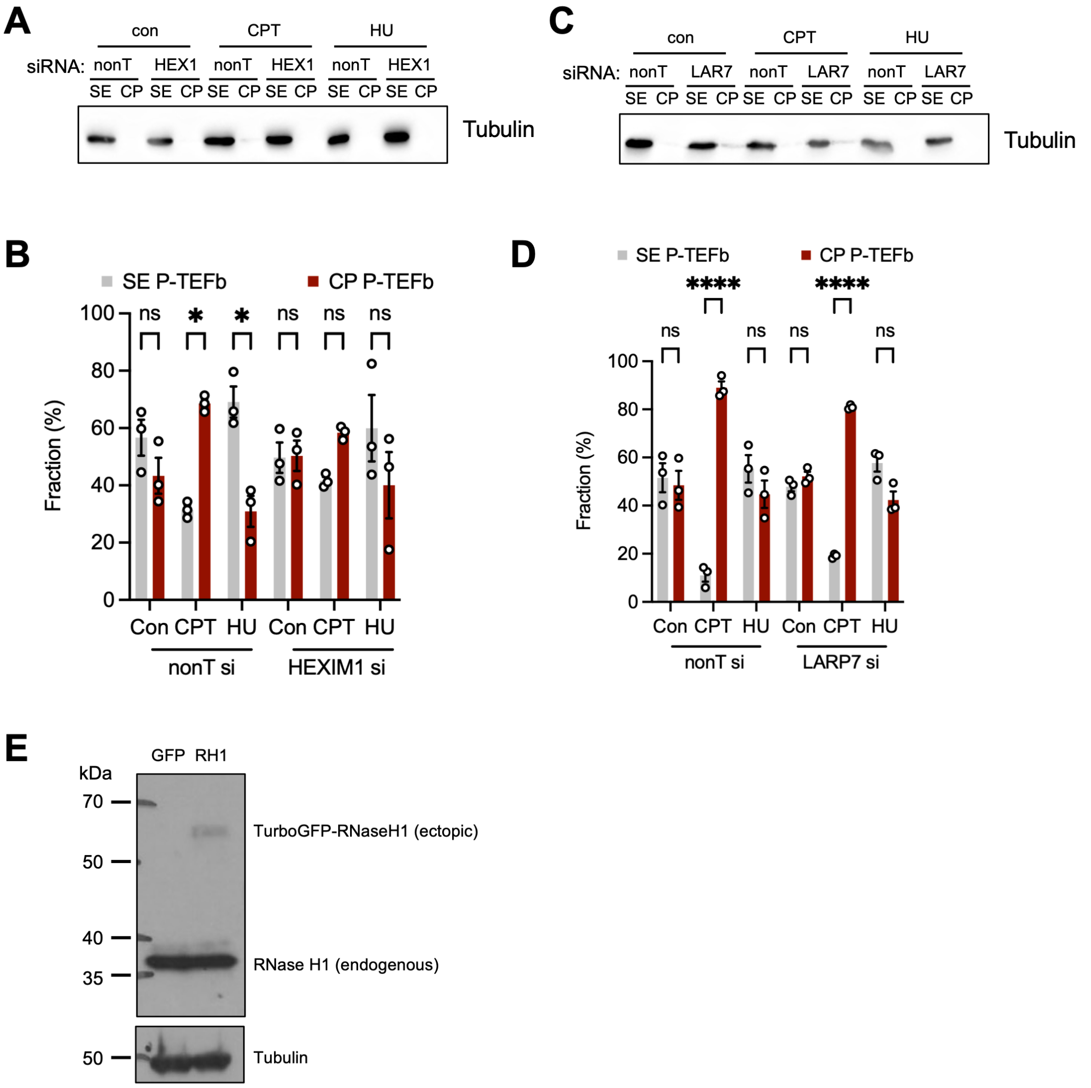

Figure S3. Impact of HEXIM1 or LARP7 depletion on P-TEFb relocation to chromatin, and ectopic RNase H1 overexpression.

A) Protein levels of Tubulin (loading control) in soluble extract (SE) and chromatin pellet (CP) fractions after 2 h treatment with 10 µM CPT or 200 µM HU +/- HEXIM1 siRNA. B) Quantification of relative CDK9 band intensity in U2OS cells treated with non-targeting (nonT) or HEXIM1 siRNA for 48 h, followed by treatment with 10 µM CPT or 200 µM HU (2 h). n=3. C) Protein levels of Tubulin (loading control) in soluble extract (SE) and chromatin pellet (CP) fractions after 2 h treatment with 10 µM CPT or 200 µM HU +/- LARP7 siRNA. D) Quantification of relative CDK9 band intensity in U2OS cells treated with non-targeting (nonT) or LARP7 siRNA for 48 h, followed by treatment with 10 µM CPT or 200 µM HU (2 h). n=3. E) Protein levels of TurboGFP-RNaseH1, RNaseH1 and Tubulin (control) in U2OS cells 24 h after control (GFP) or TurboGFP-RNaseH1 (RH1) plasmid transfection.

Column graphs show mean ± SEM. ANOVA with multiple comparisons test, * $p \leq 0.05$; **** $p \leq 0.0001$; ns: not significant. Selected p values are shown for clarity.

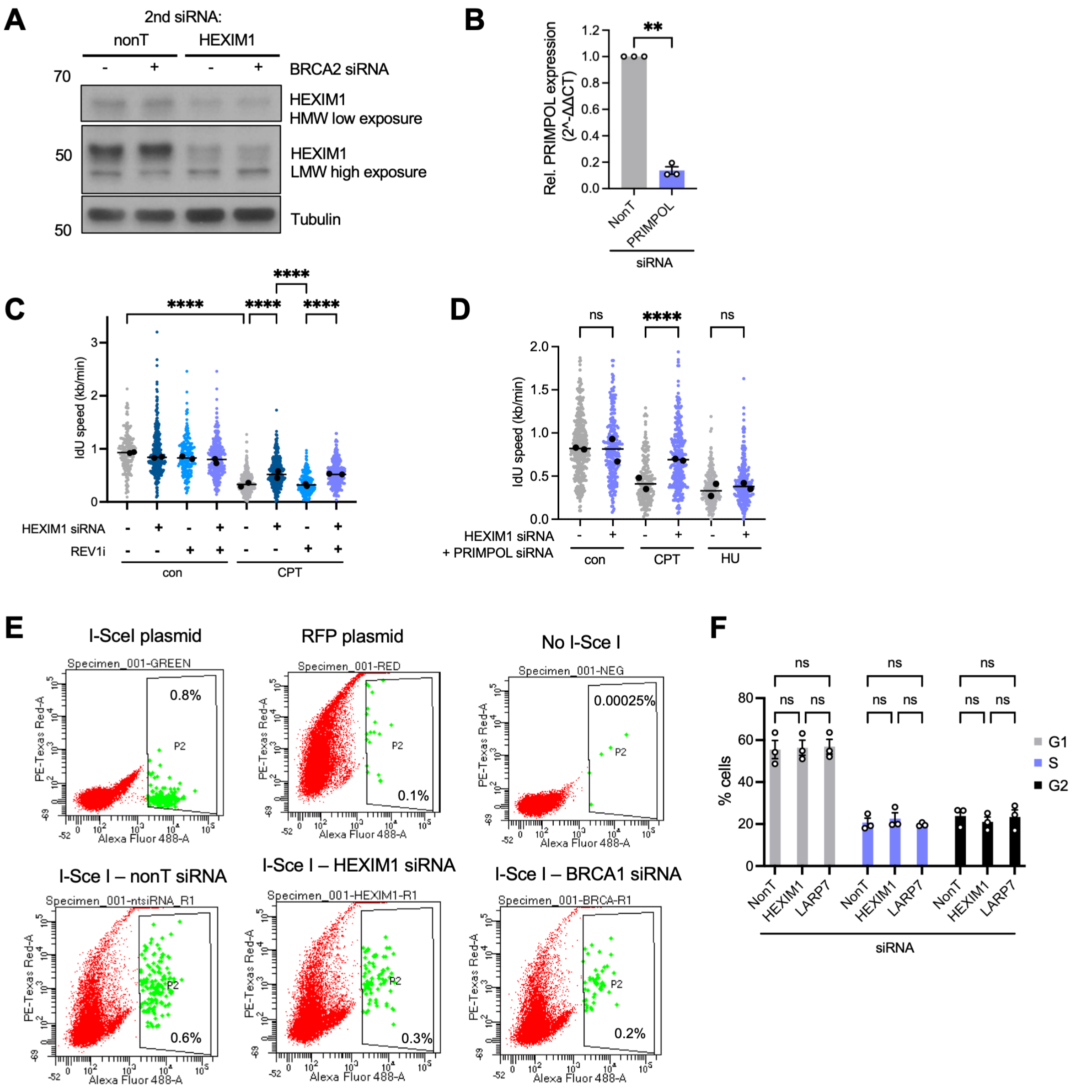

Figure S4. Role of HEXIM1 in fork reversal and homologous recombination repair.
A) Protein levels of HEXIM1 and Tubulin (loading control) in U2OS cells in presence of BRCA2 (+) or nonT (-) siRNA +/- HEXIM1 siRNA. HMW: HEXIM1 high molecular weight band, LMW: HEXIM1 low molecular weight band. B) RT-qPCR analysis of *PRIMPOL* expression in U2OS cells after 48 h treatment with non-targeting (nonT) control or PRIMPOL siRNA. n=3. C) Replication fork speeds (IdU label) after CPT treatment +/- HEXIM1 and PRIMPOL siRNA. Data from 2 repeats. D) Replication fork speeds (IdU label) after CPT treatment +/- HEXIM1 siRNA and REV1 inhibitor (REV1i). Data from 2 repeats. E) Flow cytometry gating strategy for U2OS-DR-GFP reporter assay, showing representative plots for positive controls (I-Sce I or RFP plasmids), negative control (no I-SceI transfection) and 72 h I-Sce I transfection with non-targeting (nonT), HEXIM1 or BRCA1 siRNA (96 h). F) Quantification of cell cycle distribution in U2OS cells after 72 h transfection with non-targeting (nonT), HEXIM1 or LARP7 siRNA, based on measuring DNA content using propidium iodide staining and flow cytometry. n=3.
Scatter graphs show aggregates and medians (black points) of independent repeats with overall median (line). Column graphs show mean ± SEM. Paired t-test, ANOVA or Kruskal-Wallis with multiple comparisons test, ** $p \leq 0.01$; **** $p \leq 0.0001$; ns: not significant. Selected p values are shown for clarity.

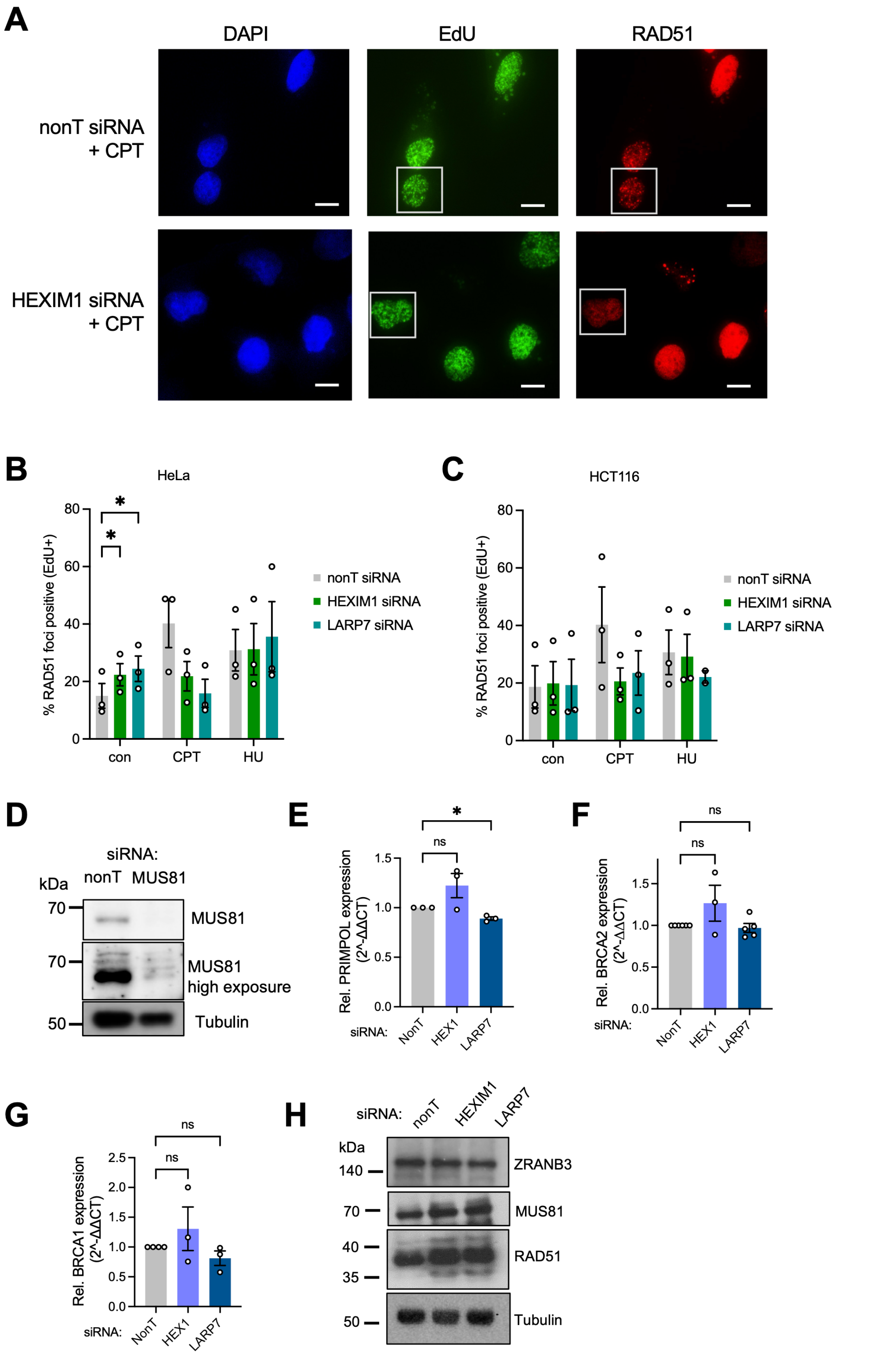

Figure S5: Impact of HEXIM1 and LARP7 on RAD51 recruitment
A) Representative images of RAD51 foci in U2OS cells after 2 h treatment with 10 mM CPT +/- HEXIM1 sRNA. Grey boxes indicate the individual nuclei shown in Figure 6A. Bars: 10 μm. B) Percentages of EdU-positive HeLa cells with RAD51 foci after 2 h treatment with 10 μM CPT

or 200 μM HU +/- HEXIM1 or LARP7 siRNA. n=3. C) Percentages of EdU-positive HCT116 cells with RAD51 foci after 2 h treatment with 10 μM CPT or 200 μM HU +/- HEXIM1 or LARP7 siRNA. n=3. D) Protein levels of MUS81 and Tubulin (loading control) 48 h after transfection with non-targeting (nonT) control or MUS81 siRNA. E) RT-qPCR analysis of *PRIMPOL* expression in U2OS cells after 48 h treatment with nonT, HEXIM1 or LARP7 siRNA. n=3. F) RT-qPCR analysis of *BRCA2* expression in U2OS cells after 48 h treatment with nonT, HEXIM1 or LARP7siRNA. n=5 (HEXIM1, n=3). G) RT-qPCR analysis of *BRCA1* expression in U2OS cells after 48 h treatment with nonT, HEXIM1 or LARP7siRNA. n=3 (nonT, n=4). H) Protein levels of ZRANB3, MUS81, RAD51 and Tubulin in U2OS cells in presence of nonT, HEXIM1 or LARP7 siRNA. Column graphs show mean ± SEM. ANOVA or mixed-effects analysis with multiple comparisons test, * $p \leq 0.05$; ** $p \leq 0.01$; *** p £ 0.001; **** $p \leq 0.0001$; ns: not significant.

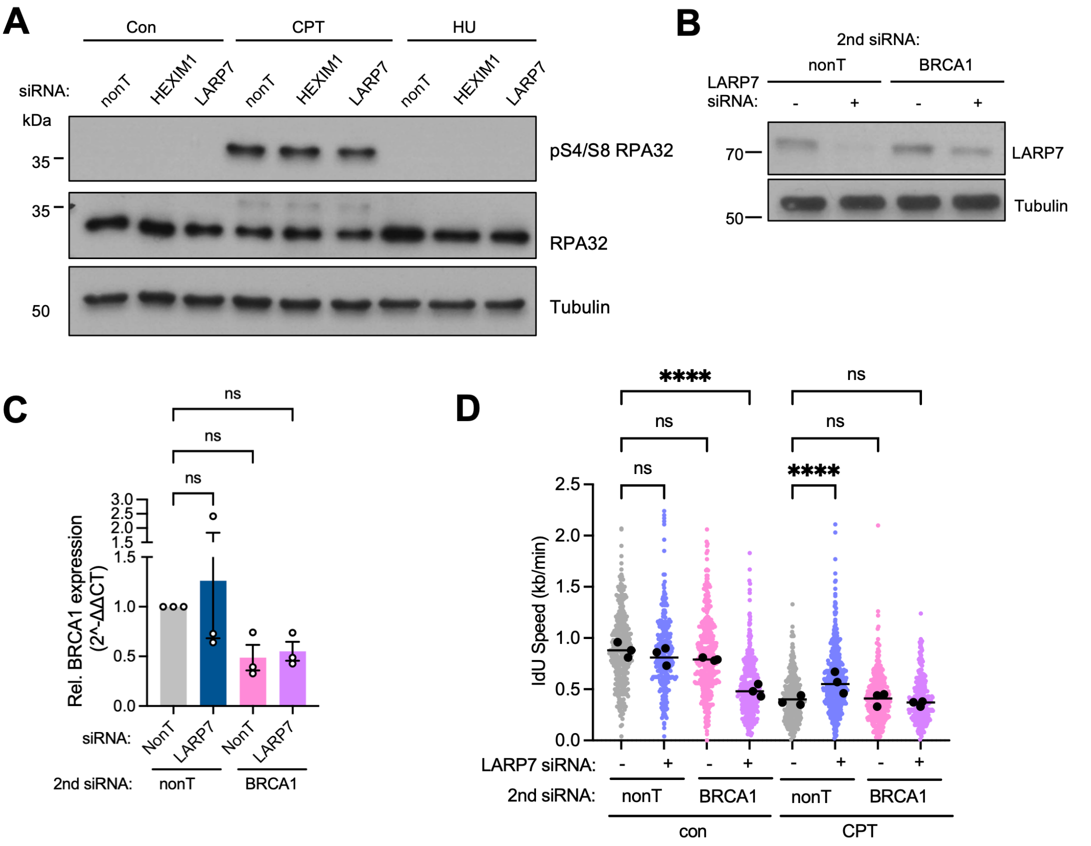

Figure S6. Roles of DNA end resection and the interaction of LARP7 with BRCA1.
A) Protein levels of phospho-serine 4/8 RPA32, RPA32 (loading control), and Tubulin (control) after 2 h treatment with 10 μM CPT or 200 μM HU +/- HEXIM1 or LARP7 siRNA. The blot shows a different experiment from that in Figure 5I. B) Protein levels of LARP7 and Tubulin (loading control) in U2OS cells 48 h after LARP7 and/or BRCA1 siRNA transfection. C) RT-qPCR analysis of *BRCA1* expression in U2OS cells after 48 h treatment with non-targeting (nonT) control or LARP7siRNA. n=3. D) Second label (IdU) speeds in U2OS cells after CPT treatment +/- BRCA1 and/or LARP7 siRNA. Data from 3 repeats.
Scatter blots show aggregates and medians (black points) of independent repeats with overall median (line). Column graphs show mean ± SEM. ANOVA or Kruskal-Wallis with multiple comparisons test, **** $p \leq 0.0001$; ns: not significant.

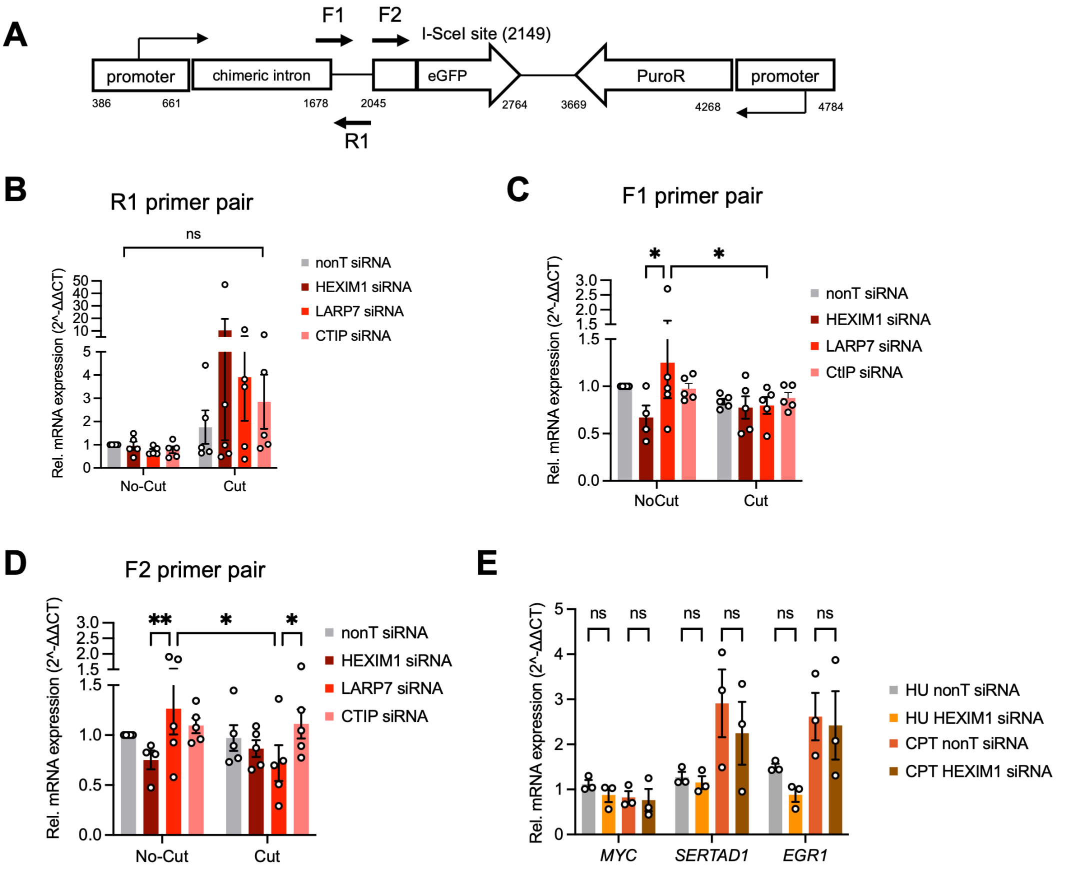

Figure S7. Impact of HEXIM1 and LARP7 depletion on transcription in the HR reporter construct.

A) Schematic of a section of the DR-GFP reporter construct with locations of eGFP and PuroR genes and their promoters near the I-SceI restriction site. Arrows show location and direction of transcripts amplified with the F1, F2 or R1 primer pairs. B) RT-qPCR analysis of transcript expression upstream of the eGFP gene in U2OS-DR-GFP cells treated with non-targeting (nonT), HEXIM1, LARP7 or CtIP siRNA for 24 h, followed by transfection (cut) or no transfection (no-cut) with RFP and I-SceI expression constructs for 72 h. n=4-5. C) RT-qPCR analysis of transcript expression at the eGFP gene in U2OS-DR-GFP cells treated as in D. n=4-5. D) RT-qPCR analysis of antisense transcript expression upstream of the eGFP gene in U2OS-DR-GFP cells treated as in D. n=5. E) RT-qPCR analysis of *MYC*, *SERTAD1* and *EGR1* expression after 2 h treatment with 200 200 μM HU or 10 μM CPT +/- HEXIM1 siRNA. n=3.

Column graphs show mean ± SEM. ANOVA with multiple comparisons test, * $p \leq 0.05$; ** $p \leq 0.01$; ns: not significant.

Supplementary Table S2. Sequence changes to generate siRNA-resistant and mutant HEXIM1 expression constructs.

Change*	Original sequence	Changed sequence
**HEXIM1 resistant to siRNA #1**	CGGAGACGTACGAGCGGTA	CCGAAACCTATGAAAGATA
**HEXIM1 resistant to siRNA #2**	AAACAGAGCCTTCGAGCTT	AAGCAATCCTTGAGAGCAT
**HEXIM1 resistant to siRNA #4**	GAGCCGAGATGTTCGCCAA	GTGCTGAAATGTTTGCTAA
**HEXIM1 PYNT to PDND**	CCCTATAACACC	CCCGATAACGAT

*siRNA #3 targets outside the coding sequence

Supplementary Table S3. Sequences of PCR primers

Name	Sequence (5' → 3')	Annealing temp (°C)
**EGR1 Forward**	TGCAGATCTCTGACCCGTTC	60
**EGR1 Reverse**	CAGGAAAAGACTCTGCGGTCA	
**SERTAD1 Forward**	GAGGACAGCCAACAAGCGAT	60
**SERTAD1 Reverse**	TGCTCAGCATCTTGCTCACTA	
**MYC Forward**	CAGCGACTCTGAGGAGGAAC	60
**MYC Reverse**	GCTGCGTAGTTGTGCTGATG	
**PRIMPOL Forward**	AAAAGCAACGTGGGGCATTC	55
**PRIMPOL Reverse**	GGTGGTTCTTCTGGCTTGGA	
**DR-GFP (F1) Forward**	GTGATCAGGCAGAGCAGGAA	58
**DR-GFP (F1) Reverse**	ACTTGTGGCCGTTTACGTCG	
**DR-GFP (F2) Forward**	TGAGCAAGGGCGAGGAGCTGTT	60
**DR-GFP (F2) Reverse**	ACACGCTGAACTTGTGGCCGTTTA	
**DR-GFP (R1) Forward**	GTCCCCTTCTCCCTCTCCAG	58
**DR-GFP (R1) Reverse**	TGAACATGGTTAGCAGAGGCT	
**BRCA1 Forward**	GCCAAGGCAAGATCTAGAGG	57
**BRCA1 Reverse**	GTTGCCAACACGAGCTGA	
**BRCA2 Forward**	CACCTCTGGAGCGGACTTATT	57
**BRCA2 Reverse**	GCTTTGTTGCAGCGTGTCTT	
**RPLP0 Forward (control)**	CAGATTGGCTACCCAACTGTT	60
**RPLP0 Reverse (control)**	GGAAGGTGTAATCCGTCTCCAC	
**ACTB Forward (control)**	TTGCGTTACACCCTTTCTTG	60
**ACTB Reverse (control)**	CACCTTCACCGTTCCAGTTT	
